# The importance of forest structure to biodiversity–productivity relationships

**DOI:** 10.1098/rsos.160521

**Published:** 2017-01-04

**Authors:** Friedrich J. Bohn, Andreas Huth

**Affiliations:** 1 Department for Ecological Modelling, Helmholtz Centre for Environmental Research GmbH—UFZ, Permoserstraße 15, 04318 Leipzig, German; 2 Institute for Environmental Systems Research, University of Osnabrück, Barbarastraße 12, 49076 Osnabrück, German; 3 German Centre for Integrative Biodiversity Research (iDiv) Halle-Jena-Leipzig, Deutscher Platz 5e, 04103 Leipzig, Germany

**Keywords:** forest stand, biodiversity, productivity, gap model, ecosystem functioning, temperate forests

## Abstract

While various relationships between productivity and biodiversity are found in forests, the processes underlying these relationships remain unclear and theory struggles to coherently explain them. In this work, we analyse diversity–productivity relationships through an examination of forest structure (described by basal area and tree height heterogeneity). We use a new modelling approach, called ‘forest factory’, which generates various forest stands and calculates their annual productivity (above-ground wood increment). Analysing approximately 300 000 forest stands, we find that mean forest productivity does not increase with species diversity. Instead forest structure emerges as the key variable. Similar patterns can be observed by analysing 5054 forest plots of the German National Forest Inventory. Furthermore, we group the forest stands into nine forest structure classes, in which we find increasing, decreasing, invariant and even bell-shaped relationships between productivity and diversity. In addition, we introduce a new index, called optimal species distribution, which describes the ratio of realized to the maximal possible productivity (by shuffling species identities). The optimal species distribution and forest structure indices explain the obtained productivity values quite well (*R*^2^ between 0.7 and 0.95), whereby the influence of these attributes varies within the nine forest structure classes.

## 1. Introduction

Human activities alter ecosystems and their functions [1]. One important function of ecosystems pertains to their productivity. Many biodiversity experiments show higher levels of productivity in species-rich ecosystems than in monocultures (e.g. [2–4]). Nevertheless, the generality (e.g. in forests) of a positive relationship between biodiversity and productivity is still debated because most of the results are derived from grassland experiments [5].

In recent years, an increasing number of biodiversity experiments have been designed for forests. However, trees examined in these experiments have often been very young (e.g. [6,7]). As an alternative method, inventories of natural and managed forests have been analysed. Several field studies of forests have revealed positive effects of biodiversity on productivity (e.g. [8–13]), but others have found invariant or even negative relationships (e.g. [14–16]). A meta-analysis of published field measurements reveals equal abundances of species and a broad variety of light strategies among species as important prerequisites for positive biodiversity–productivity relationships [17].

In addition to field studies and experiments, forest models serve as a means of investigating biodiversity–productivity relationships. They allow the analysis of a variety of species mixtures over long periods. Morin *et al*. [18] used a well-established forest gap model [19] to show that tree species richness promotes productivity (defined as biomass increments per year) in mature European temperate forests. In addition, the authors show that competition for one resource (here light) is sufficient to generate an increase in forest productivity with species richness.

Despite the widely recognized positive trends found in diversity–productivity relationships in forests, it remains unclear why different relationships can be found. Besides the influence of species diversity it is well known that basal area also influences productivity (e.g. [12]). Recent studies also highlight the influence of tree size heterogeneity on productivity. For instance, some studies found a negative effect of size heterogeneity in several monocultures [20,21], whereas Dănescu *et al*. [22] revealed positive effects of size heterogeneity (and species diversity) in mixed stands.

In this study, we analyse the role of species diversity and forest structure, which is described by basal area and tree height heterogeneity, on forest productivity. We focus on small-scale forest stands (400 m^2^), wherein all trees of a given structure interact due to light competition. Every forest stand is characterized by its tree species mixture and forest structure. We simulated a large set of forest stands by randomizing species identities using a new forest modelling approach, which we refer to as the forest factory approach. The range of investigated forest types includes natural, disturbed and managed forests as well as young and old forests. We estimate the wood production of every tree in the stand using physiological relationships encoded in a process-based forest model. The wood production of all trees in the analysed stands is summed up to estimate instantaneous above-ground wood production [23]. This approach also allows an exploration of the effect of species distribution within a given forest structure. We suggest a new index for analysing this effect.

Using this new forest factory approach, we analyse approximately 300 000 forests stands to answer the following questions: (i) How does forest structure compared with species number influence forest productivity (AWP)? How does especially tree height heterogeneity influence forest productivity? (ii) Do different forest structure types show different biodiversity–productivity relationships? (iii) How does basal area and tree height heterogeneity influence forest productivity compared with optimal species distribution?

## 2. Material and methods

### 2.1. Overview of our approach

**Figure 1.**
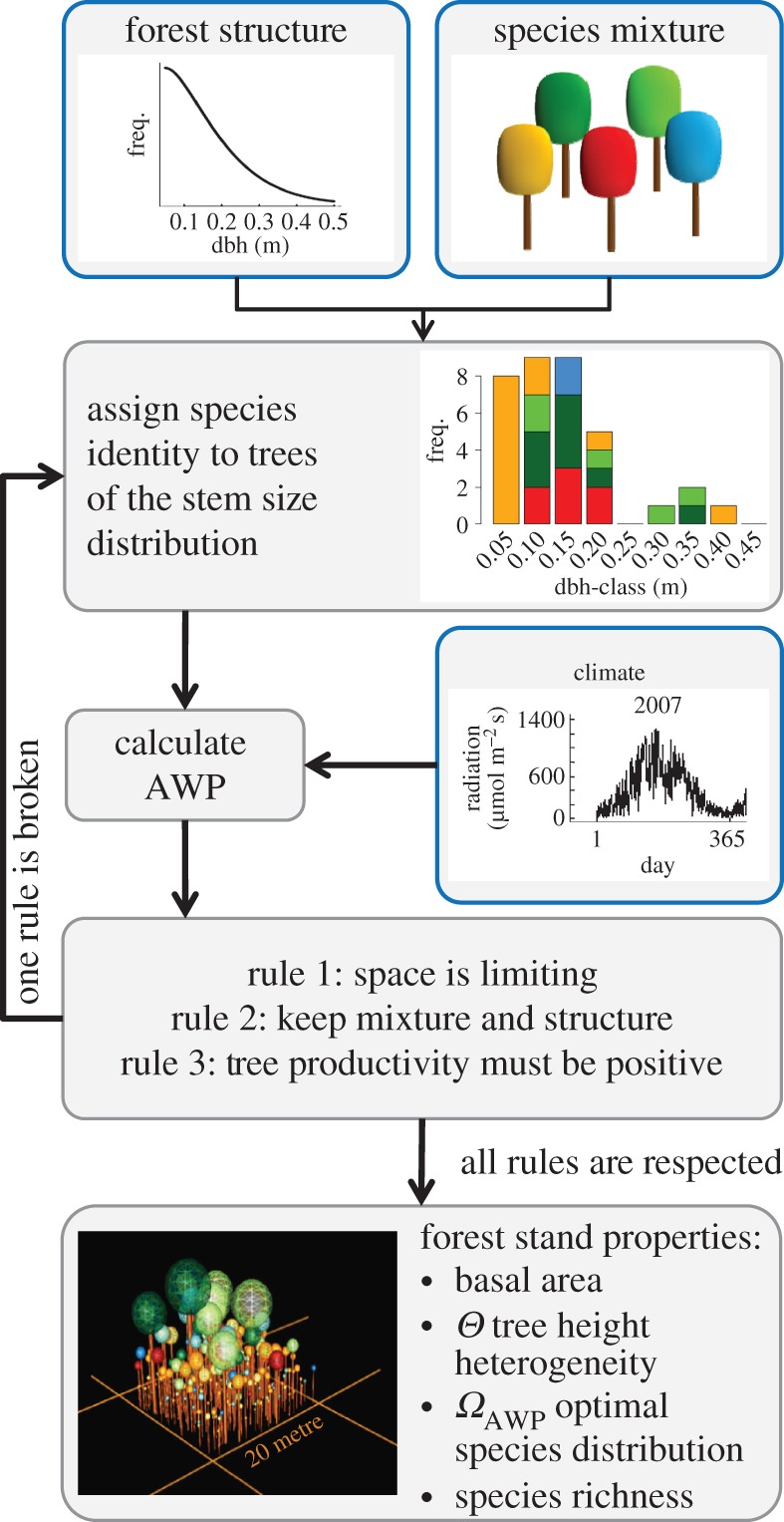
Workflow to generate one forest stand with the forest factory approach. Blue boxes indicate the input variables.

The forest factory method involves generating a large number of forest stands that vary in their tree species mixtures and forest structures (figure 1). The creation of forest stands is based on three simple construction rules (see §2.3). Forest stands are formed based on different stem diameter distributions, which represent typical forest structures of early to late succession and of managed and unmanaged temperate forests. Species mixtures include the following eight species: pine, spruce, beech, ash, oak, poplar, robinia and birch (255 mixtures in total). These species vary markedly in shade-tolerance, allometry, productivity and responses to climate (more information is included in electronic supplementary material, appendix C). Based on environmental conditions, tree productivity is measured for each single tree over 1 year (see §2.2) and is summed. We then group forest stands into nine different forest structure classes based on basal area and tree height heterogeneity values (see §2.4). Note that we only simulate individual years and do not simulate forest succession over longer time periods (e.g. hundreds of years). Finally, we quantify the influence of forest properties (basal area, tree height heterogeneity and optimal species distribution) on forest productivity (see §2.5).

### 2.2. Productivity of a single tree

We use processes of a spatially explicit forest gap model (here FORMIND) to estimate tree productivity (above-ground wood production, AWP). Over the last 20 years, this model has been used to study various forests around the world (e.g. [24–30]). The model simulates establishment, mortality, growth of trees including competition for light. Eight tree species have been parametrized for the temperate zone differing in their shade-tolerance, allometric relationships, carbon allocation processes and preferred environmental conditions. In a previous study [23], these were parametrized using forest yield tables for Germany and measured species-specific traits and were validated using yield tables for France.

The forest factory method only uses competition and productivity processes of the forest gap model as well as allometric relationships. The allometric relationships used are assumed to be invariant (independent of the competitive environment a tree is found in). However, productivity rates of single trees (AWP_tree_) are affected by available light, available soil water and air temperature. To estimate these tree-specific environmental conditions, we use daily mean radiation, daily mean air temperature and daily sum of precipitation (1 year simulation). The available light at the top of a tree is the measured radiation level reduced by the shading due to larger trees within a forest stand. The available soil water within a stand results from precipitation, tree evapotranspiration and run-off. We used a climate dataset from a temperate forest in central Germany (eddy flux station at Hainich in the year 2007 [31]; electronic supplementary material, appendix A.1, figure A1).

The productivity rate of a single tree is calculated as the difference between photosynthetic production and respiration. The calculation of photosynthetic production uses species-specific light-response curves and includes the available light at the top of a tree, crown size and self-shading with its crown. In addition, limitations due to available soil water and air temperature can modify the productivity rate [23,27,28].

The photosynthesis of a tree (*P*_tree_) is partly consumed by its maintenance respiration *R*_m_, which is dependent on air temperature and tree biomass [32], including allocation to non-wood tree tissues. Remaining organic carbon is transformed for above-ground wood production (AWP_tree_ and into a proportional fraction through growth respiration (*r*_g_)).

$$\mathrm{AW}P_{\mathrm{tree}}=(P_{\mathrm{tree}}-R_{m})\times (1-r_{g}).$$

To get the forest stand AWP, we sum up AWP_tree_ of all trees of the forest stand. Note that changes of AWP due to mortality and establishment processes within a forest stand are not included [33].

### 2.3. Construction of forest stands

**Figure 2.**
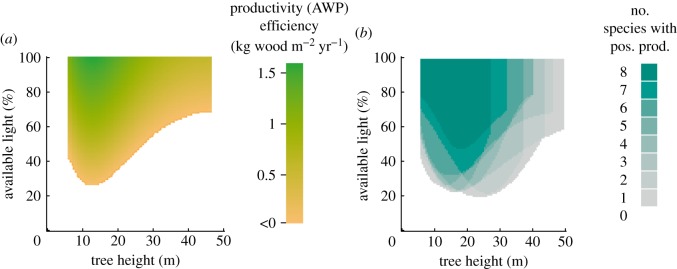
(*a*) Productivity efficiency (AWP_tree_ per unit crown area of pine) depends on tree height and available light at the top of a tree under the given environmental conditions at the Hainich station in the year 2007. Productivity efficiencies of tree heights with a diameter at breast height (dbh) smaller than 5 cm and light–height combinations with negative productivity are not plotted (white area). (*b*) The pancake plot shows how many species exhibit positive productivity under certain light–height conditions.

Forest stands vary in forest structure and species mixture and each cover an area of 400 m^2^. The creation of forest stands follows three construction rules: (i) Available space limits the number of trees within a forest stand. Thus, the spatially explicit modelled shape of tree crowns limits the maximum number of trees per area. (ii) A predefined forest structure and species mixture is realized. Forest structures are often described based on stem size distributions, which follow a Weibull distribution (e.g. [34–36]). We use 15 different stem size distributions to describe even-aged (trees with the same stem diameter) and uneven-aged forests (variable stem diameters) of different successional states (all distributions are shown in detail in electronic supplementary material, appendix A.2, figure A3). The stem diameters range between 5 and 50 cm. However, (iii) every tree must exhibit positive productivity. The productivity of a tree is dependent on its species identity, species-specific limitation factors of photosynthesis, tree height and degree of shading from larger trees. As we always use the same climate dataset for construction, photosynthesis limitation factors are constant but species-specific (figure 2*a* shows productivity for pine while those for other species are shown in electronic supplementary material, appendix A.2, figure A4). Productivity levels under the same environmental conditions vary among species, as does the number of possible combinations of light and height levels, which result in positive productivity (figure 2*b*: areas of positive productivity for all species). As a tree must maintain positive productivity to survive, there are light–height conditions in which some species cannot survive. In such cases, the species identity of a tree (with negative productivity) is replaced by an alternative species identity of the existing mixture, which shows positive productivity. In some cases, when no species identity allows for positive tree productivity, the number of trees is reduced. The presence of fewer trees reduces the shading levels such that one species identity with positive productivity can also be found for shaded trees (for a more detailed account of the creation of forest stands, see electronic supplementary material, appendix A.3).

In total, we generated 379 170 forest stands. The generated forest stands reach a basal area of up to 60 m^2^ and an above-ground biomass of 543 t odm ha^−1^. Stem quantities range from 25 to 14 000 stems per hectare.

### 2.4. Forest productivity and classification

The analysed productivity of forest stands represents the mean above-ground production over 5 years. Each year is simulated separately using the same initial forest stand (to simulate forest stand productivity under variable climates; see electronic supplementary material, appendix A.1, figure A2). We used climate data of the Hainich station climate data for the years 2000–2004. It should be noted that the new method used in this study requires much less computation time than do normal forest succession simulations because only a few years need to be simulated for each forest stand to obtain productivity.

We use two simple measures to quantify the structure of forest stands: basal area (BA), which is the sum of all cross sections of stems at breast height per area, and the standard deviation of tree heights within a forest stand to describe tree height heterogeneity (*Θ*). For further analysis, we grouped the dataset into nine structure classes: three basal area classes (small, moderate and large, 5–15, 15–25 and 25–35 m^2^ ha^−1^, respectively) and three tree height heterogeneity classes (low, moderate and high, 0.5–2.5, 2.5–4.5 and 4.5–6.5 m, respectively).

**Figure 3.**
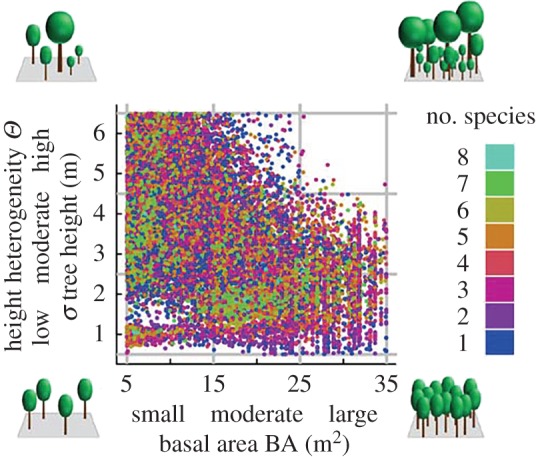
Overview of the 300 000 selected forest stands. Scatterplot between basal area and tree height heterogeneity, where each dot represents one forest stand. Colours denote the number of species within a forest stand. For the sake of clarity, we randomly select 3000 plots for every species quantity (1–7 species) and all forest stands including eight species. Grey lines separate the nine different structure classes of low, moderate and high (0.5–2.5, 2.5–4.5 and 4.5–6.5 m) tree height heterogeneity *Θ* levels and small, moderate and large (5–15, 15–25 and 25–35 m^2^ ha^−1^) basal areas. The four smaller pictures in the four corners serve as illustrations of the forest structure classes of the forest stands.

Nearly all structure classes contain forest stands, which include between one and eight species. Only the forest stand with a high BA and high *Θ* cover a range from one to seven species (figure 3; electronic supplementary material, appendix A.3, figure A5), which contains only 0.4% of all forest stands. This is a result of random construction processes within the forest factory. Note that the maximum species number of possible species has been assumed to be eight in all cases. The forest stands which belong to one of the nine classes sum up to 299 669 forest stands.

For all stands within the same structure class and with the same species mixture we determine an average productivity (AWP_mixture_). Using this method, all mixtures have the same weight in the structure classes. We then average the AWP_mixture_ of forest stands, which include the same number of species to obtain an average productivity (AWP*_n,s_*) as a function of the species number for each structure class (figure 6).

There are two ways to analyse aggregate productivity values. First, we can average productivity values for different species quantities while keeping the structure constant (figure 5*a*).

$$\mathrm{AW}P_{S}=\frac{1}{n}\overset{n}{\underset{i=1}{\sum }}\mathrm{AW}P_{s,i}.$$

In the same way, we can calculate the average productivity level for different structure classes while keeping the species number constant (figure 5*b*).

$$\mathrm{AW}P_{N}=\frac{1}{s}\overset{s}{\underset{i=1}{\sum }}\mathrm{AW}P_{i,n}.$$

### 2.5. Quantification of the influence of forest properties on forest productivity

Forest productivity is dependent on basal area. Thus, the presence of a larger number of trees of the same size increases AWP. Second, AWP is dependent on the vertical tree size structure that we quantified by tree height heterogeneity (*Θ*). Two trees, which do not shade each other, have a higher AWP than the same trees, where one tree shades the other.

**Figure 4.**
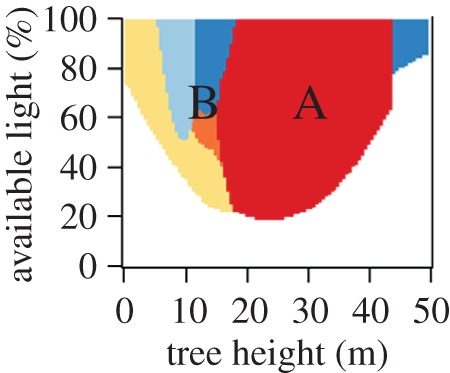
The optimal species distribution plot shows under which conditions (tree height and available light) species present maximum productivity per crown area based on environmental conditions at the Hainich station in the year 2007. Trees in a forest of tall trees of moderate tree height heterogeneity should be found around A, whereas trees in a forest of moderately sized trees and moderate tree height heterogeneity should be found around B. Colours denote different species (light blue, pine; blue, spruce; yellow, robinia; orange, ash; red, beech).

Beside basal area and tree height heterogeneity we propose a third forest property index, which we refer to as optimal species distribution in relation to AWP (*Ω*_AWP_). Here, we analyse AWP changes due to a change in species identity. For instance, a forest with large beech trees that cover small birch trees has another AWP compared with a forest with birch trees covering beech trees, although the forest structure (and environmental conditions) stays the same. We suggest quantifying this effect by

$$\Omega_{\mathrm{AWP}}=\frac{\mathrm{AW}P_{\mathrm{obs}}}{\mathrm{AW}P_{\max}},$$

with the observed forest productivity and with the maximum possible AWP of a given forest structure. In calculating, we determine those species identities for every tree found in a forest, which show the maximal AWP (figure 2). Depending on the forest structure, we found compositions of optimal species identities assigned to trees in the forest. For instance, a forest with tall trees and moderate tree height heterogeneity shows optimal AWP if it only consists of beech trees (area A in figure 4). In a forest with moderately sized trees and moderate tree height heterogeneity, different species can exhibit optimal AWP (area B in figure 4). To quantify the importance of these three mechanisms, we made a partitioning of the variance using as explanatory variable: tree height heterogeneity, basal area and *Ω*_AWP_.

### 2.6. Analysis of the German forest inventory

To compare our results with German forest inventory III (2012), we selected forest plots that (i) host only species considered in this study and (ii) are located on flat terrain (sloped at less than 15%). As the inventory is based on variable radius sampling, we only considered plots with trees with a maximum diameter at breast height (dbh) of 0.5 m (which results in a maximal area of approx. 400 m^2^ of the plots).

We analysed the influence of structure on productivity within the selected plots of the German forest inventory in the same manner as the forest factory and calculated first AWP_mixture_ (with a minimum of two plots per class of every species composition). In total, 5054 forest plots of the German forest inventory were processed. We then calculated $\mathrm{AW}P_{S}$ and $\mathrm{AW}P_{N}$ (see above). Because forest stands with large tree height heterogeneities were rare (2% show height heterogeneity larger 4 m), we calculated results for four new height heterogeneity classes (0–1, 1–2, 2–3, 3–4 m) and added one basal area class (35–45 m^2^). A comparable analysis using forest stands of the forest factory uses the same structure classes. Further, we quantify the effect of diversity on productivity by averaging over all structure classes (results in electronic supplementary material, appendix B.2).

## 3. Results

In this study, forest stands, generated via the forest factory approach, are used to investigate the effects of forest structures and biodiversity levels on productivity (AWP). We classify forest stands into nine different forest structure classes that differ in tree height heterogeneity and basal area (in total, approx. 300 000 forest stands). The tree species diversity levels range from one to eight species.

### 3.1. Analysis of the forest stands of the forest factory

**Figure 5.**
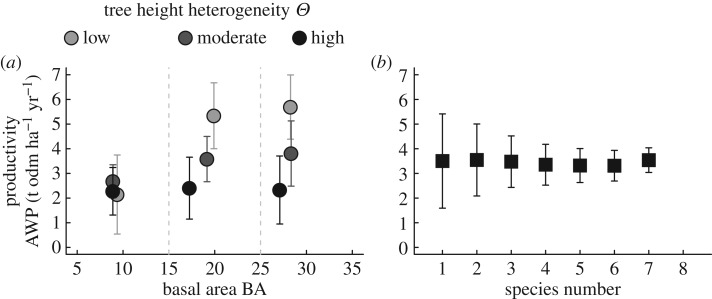
Analysis of mean productivity (above-ground wood production) of the forest stands. (*a*) Mean productivity of the nine structure classes: small, moderate and large basal area BA (5–15; 15–25; 25–35 m^2 ^ha^−1^) and low, moderate and high tree height heterogeneity *Θ* (0.5–2.5; 2.5–4.5; 4.5–6.5 m); (*b*) mean productivity depending on the numbers of species in a forest stand. Grey bars denote the mean standard deviation.

The mean productivity of the nine forest structure classes varies from 2.1 to 5.7 t organic dry matter (odm) ha^−1^ yr^−1^ (figure 5*a*). Increasing basal area results in increasing forest stand productivity in cases of low and moderate height heterogeneity. Height heterogeneity is negatively correlated with productivity for stands with moderate and large basal areas. Species richness hardly influences the forest productivity, which remains relatively constant at 3.5 t odm ha^−1^ yr^−1^ (figure 5*b*). In contrast with the effect of structure, variability of productivity is negatively affected by species richness. This observed effect of structure or diversity on productivity does not change when functional diversity is analysed instead of species number (electronic supplementary material, appendix B.1, figure B1). We also analysed the sensitivity of these results to an increase or decrease in the mean annual temperature of 1.5°C. Here, the absolute productivity changes slightly but the general pattern remains the same (see electronic supplementary material, appendix B.1, figure B1).

**Figure 6.**
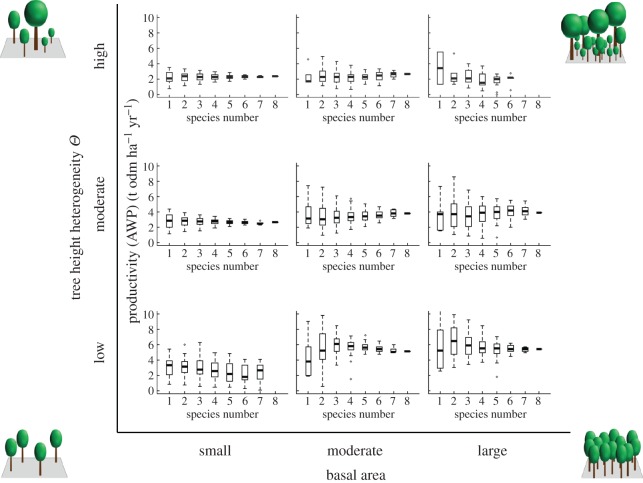
Boxplots of productivity values (AWP) for each number of species among the nine forest structure classes: small, moderate and large basal areas BA (5–15; 15–25; 25–35 m^2 ^ha^−1^) and low, moderate and high tree height heterogeneity *Θ* (0.5–2.5; 2.5–4.5; 4.5–6.5 m). The four smaller pictures in the four corners illustrate the forest structure classes of the forest stands.

When analysing the relationship between tree diversity and productivity for forest stands for each structural class, we found several relationships (figure 6): (i) increasing relationships for forest stands with moderate or large BA and moderate *Θ*; (ii) a bell-shaped relationship for forest stands with moderate or large BA and low *Θ*; (iii) invariant relationships for forest stands with small BA and moderate or high *Θ* and (iv) decreasing relationships for forest stands with small BA and high *Θ*. In nearly all cases, the variability of productivity decreases with increasing richness. Overall, from low to high basal area and from high to low tree height heterogeneity increases the effect of species numbers on the diversity–productivity relationship (mean productivity and variability). However, the effect of diversity on productivity among classes is less pronounced than the general effects of structure (figure 5*a* and figure 6).

### 3.2. Quantification of the influence of forest properties on forest productivity

**Figure 7.**
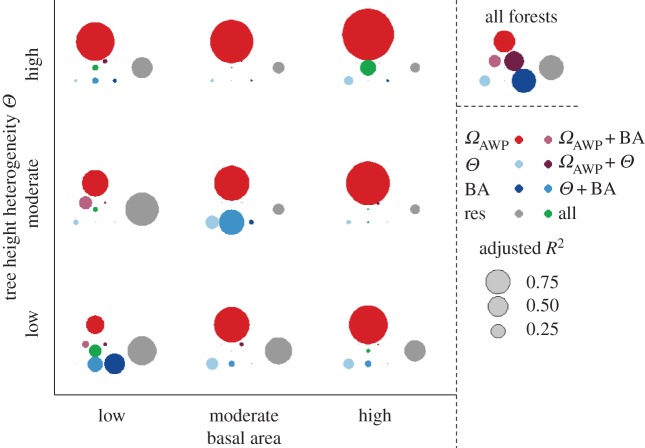
Partition of variance for all forests and for the nine structure classes. Circles denote the adjusted *R*^2^. Colours represent the different mechanisms (blue, forest structure; red, *Ω*_AWP_ purple, additional *R*^2^ for combinations of *Ω*_AWP_ and one structure mechanism; green, additional *R*^2^ when all mechanisms are combined).

The three forest properties (basal area, tree height heterogeneity and *Ω*_AWP_) influence forest productivity quite strongly but differing in their intensity for different forest classes. For all forest stands, the three mechanisms together explain 70% of the variance, with BA and *Θ* alone explaining 50% (figure 7; all forests). For eight of nine classes, *Ω*_AWP_ is the most important driver of productivity. Only forests presenting low height heterogeneity small basal areas are more sensitive to structural changes than *Ω*_AWP_. With increasing basal area and tree height heterogeneity, *Ω*_AWP_ becomes increasingly important. Species richness is weakly correlated with the three mechanisms (*R*^2^ values up to 0.25, see also electronic supplementary material, appendix B.5, figure B8). Note that a high correlation between forest properties (BA, *Θ* and *Ω*_AWP_) and productivity does not automatically correspond to a high correlation between such mechanisms and diversity. For instance, in forest stands of low height heterogeneity and large basal area, diversity correlates best with tree height heterogeneity (*R*^2^ = 0.23), but productivity is mainly determined by *Ω*_AWP_ (*R*^2^ = 0.75), which is not correlated with diversity (*R*^2^ = 0.01; see electronic supplementary material, appendix B.5, figure B8)

### 3.3. Analysis of the German forest inventory

**Figure 8.**
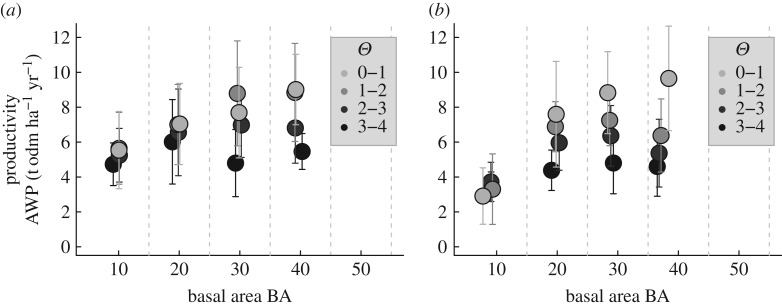
Analysis of mean forest stand productivity (above-ground wood production) of the German forest inventory (*a*) and forest stands of the forest factory (*b*) for 16 structure classes. Basal area classes were 5–15, 15–25, 25–35 and 35–45 m^2 ^ha^−1^. Tree height heterogeneity (*Θ*) classes were 0–1, 1–2, 2–3 and 3–4 m.

We analysed the German forest inventory with the same method as applied to the forest factory. The productivity (AWP) of forest increases with basal area, whereas it decreases with increasing tree height heterogeneity. This pattern is quite similar to the pattern observed in the analysis of the corresponding subsample of the forest factory dataset (figure 8). However, the influence of tree height heterogeneity on forest productivity is higher in the forest factory dataset. For forest stands with high *Θ*, we observe almost constant productivity of forest stands as in the forest stands of the forest factory. According to our analysis, diversity detached from forest structure shows no effect on productivity in the German forest inventory (for further details, see electronic supplementary material, appendix B.2).

## 4. Discussion

In this study, we presented a new approach (the forest factory method), which allows us to investigate the interplay between structure, tree species diversity and productivity in forest ecosystems. We analysed 300 000 forest stands generated by the forest factory model approach, whose constructions are based on forest gap model algorithms including species-specific tree allometries, growth processes and inter-individual competition. The analysis revealed strong effects of forest structure on productivity compared with effects of diversity. For different structure classes, we found increasing, constant, decreasing and bell-shaped relationships between species richness and forest productivity.

### 4.1. The forest factory approach

The forest factory method applies typical algorithms used in forest models to simulate annual forest growth for a large number of forest stands. The construction of forest stands follows simple rules. Using this method one does not need to simulate forest succession over long periods (e.g. hundreds of years) and hence the computation time required for this approach is quite short. This constitutes an important advantage, as computation time is a strong limiting factor for classical studies that use forest models [37]. Our approach covers a broad set of successional stages of managed, disturbed and natural forests. We assumed an equal weight for each constructed forest stand. However, it would be possible to include information about frequency of forest stands to adapt the sampling to that of a certain field study. For instance, the German forest inventory includes 20 times more forest stands with *Θ* < 1 m compared with stands with *Θ* > 4 m.

Allometric relationships, modelled tree growth and competition processes behind this analysis have been tested and applied in several studies. Nevertheless, one should be aware of several constraints. For instance, we use average species-specific allometries, which are based on yield tables of monocultures and average values of measured traits [23]. The allometries could differ from those of a specific site, as they can be influenced by the individual growth conditions of trees [13]. In addition, disturbances and management can modify individual tree allometries. Including allometric processes measures in forest models is an important, but also complex task and should be considered in future work. Please note that the model used does not consider competition for nutrients. According to Zhang *et al*. [17], this factor might be of limited relevance to diversity–productivity relationships (because nutrient supplies do not typically change considerably over a few metres and within 1 year).

One important rule for the construction of forest stands is that trees must exhibit positive productivity (third rule of the forest factory). However, trees can also survive for several years in poor conditions. Therefore, in our final analysis, we determined mean productivity levels for five individual years. Thus, our results are not affected by one specific year. They represent the average productivity level for a temperate climate including years characterized by poor and good growing conditions. However, when climatic patterns change for a longer period of time, all species will change their productivity pattern (under the conditions of available light and tree size, figure 2*b*). A second important point in the forest factory approach is that shade-tolerant trees have a higher probability to occur in the understorey, as these shade-tolerant trees grow also under reduced light conditions. This higher abundance of shade-tolerant species is also found in field studies [38]. It is unclear how relevant this effect is within forest stands of the forest factory We, therefore, run an additional analysis to quantify the influence of unequal abundances using only forest stand with more or less equal abundance (*F*_Eve_ greater than 0.9; [39], see electronic supplementary material, appendix B.6, figure B10). We found similar patterns as in the original analysis. Only forests with high basal area and high tree height heterogeneity differ in their average productivity from the analysis of the full dataset. In these forests, we found a lower AWP (compared with the full dataset).

In this study, we focus on competition between trees at small spatial scale over short time periods. For larger forests (e.g. several hectares) structural characteristics can differ from small-scale features. For instance, for an old-growth forest, height heterogeneity levels may be quite high if the forest includes small and large trees. Nevertheless, trees could be divided into small-scale regeneration patches with small trees and sections that include mainly large trees. Such a forest would exhibit productivity levels which can be estimated from productivity levels over several forest stands with low tree height heterogeneity. This productivity would be higher (all patches have a low *Θ*) than productivity of a forest consisting of patches including large and small trees on a local scale (all patches have a high *Θ*; figure 5*a*). The same consideration could be made for longer time periods (where mean *Θ* is high, whereas for different points in time the *Θ* of forest stands could be quite low).

### 4.2. Comparisons with field datasets

Our analysis of the forest factory and the analysis of the German forest inventory show that variables, which characterize forest structure (basal area, tree height heterogeneity), are the dominating drivers of forest productivity for the analysed large datasets (figure 7 and 8). Analysis of other national forest inventories found also strong effects of forest structure. For instance, Vilà *et al*. [12] analyse six European forest inventories (the German inventory was not included) and identify basal area as the most important variable to predict forest productivity, whereas diversity was of minor importance. Paquette & Messier [10] found similar results analysing 15 000 plots of the Canadian forest inventory. However, tree size heterogeneity was not included in these studies. Bourdier *et al*. [21] analyse stands of the French forest inventory (approx. 6000 plots) with one species and found a negative effect of tree size heterogeneity on forest productivity. However, other analysis reveals a positive effect of tree height heterogeneity. For instance, Dănescu *et al*. [22] analyse approximately 400 plots (which vary in time and/or location) in southern Germany covering a broad climate gradient. The plots host different mixtures of three species. The positive effect of tree size heterogeneity might be related to the larger size of the analysed stands (plot area ≥ 0.27 ha). As discussed above, such large area could be a composition of small stands with different forest structures (which may favour higher productivity than a homogeneous structured stand). We also showed that forests with larger height heterogeneity are quite sensitive to optimal species distribution. With increasing *Ω*_AWP_ forest productivity rises. This seems to outperform the negative effect of height heterogeneity in the stands in southern Germany.

Our analysis of the forest factory dataset (and the German forest inventory) shows no effect of diversity on forest productivity (figure 5; electronic supplementary material, appendix B.2). However, when the mean productivity for different diversities was calculated (and forest structure was not considered), we found a 10% increase in productivity between one and two species mixtures within the German forest inventory. This corresponds to the results of other studies (e.g. [9], Catalonian forest inventory, [17]). This positive relationship may be attributed to the fact that 71% of German forest inventory plots show a *Θ* lower than 2.5 m and basal area values greater than 25 m^2^ ha^−1^. These types of forests, which are mainly driven by the optimal-species-distribution mechanism, show positive relationships between diversity and productivity (figure 7). In electronic supplementary material, appendix B.8, we discuss additional studies that found positive (e.g. [8]) and negative effects of diversity on productivity (e.g. [15]) with our results.

### 4.3. Forest properties

Additive partitioning can be used to analyse the influence of complementarity and selection effects on diversity–productivity relationships [3]. This method is based on a comparison of observed yields with expected yields (see electronic supplementary material, appendix B.4 for further details). For our analysis using 300 000 forest stands, no significant trends were found concerning complementarity and selection effects (the best *R*^2^ is 0.01; see electronic supplementary material, appendix B.4, Figure B6). Also for forest stands in the different structure classes we got similar results; both factors could not explain productivity variations (the best *R*^2^ is 0.06). Forest properties (basal area, tree height heterogeneity and *Ω*_AWP_) explain forest productivity much better (figure 7).

Different indices were developed to describe forest structure (e.g. [40]). We tested also the effect of mean tree height (by replacing basal area in the analysis, see electronic supplementary material, appendix B.7, figure B11). We observed similar patterns compared with the original analysis. This might be related to the fact that for the stands of the forest factory basal area and mean height are partly correlated (*R*^2^ = 0.51). However, it would be worth analysing the influence of additional structural indices and combination of them on the diversity–productivity relationships (e.g. LAI, Gini index of tree sizes, stem density index).

## 5. Conclusion

In this study, we present a novel approach (the forest factory method), which generates possible combinations of forest structures and species mixtures. We show that over a broad range of forest stands, forest structures are the dominant drivers of forest productivity. However, subsamples reveal various diversity–productivity relationships that can be related to forest structure indices or optimal species distribution.

## Supplementary Material

Appendix A: Additional information regarding method and validation

## Supplementary Material

Appendix B: Additional information regarding results and discussion

## Supplementary Material

Appendix C: detailed Model describtion

## Data accessibility

The dataset supporting this article has been uploaded to the Dryad Digital Repository: http://dx.doi.org/10.5061/dryad.88j7k [41].
