# Supplementary material for: The importance of forest structure to biodiversity–productivity relationships
Source: R Soc Open Sci. 2017 Jan 4;4(1):160521. doi: 10.1098/rsos.160521 (PMC5319316; doi:10.1098/rsos.160521)
Supplement: Appendix A: Additional information regarding method and validation [file rsos160521supp1.pdf]

Appendix A - Additional information regarding method and validation

A.1 Climate data

Two climate time series are used in this study. The first one (figure A1) consists of one "typical" year and it is needed in the forest factory for the creation process of the forest stands. The second one (figure A2) consists of five years, which are used to calculated average productivities of these five independent scenario years. Note, tree allometries stay constant for all five scenarios.

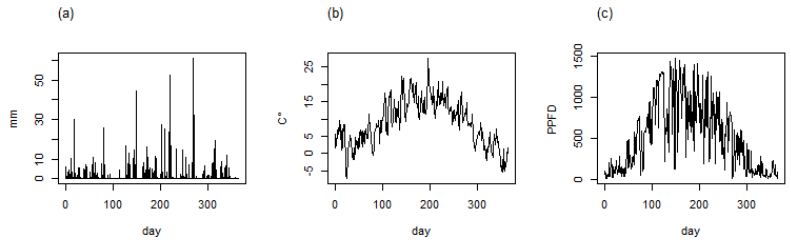

Figure A1: Overview of climate conditions used as input for the forest stand creation. The climate data set was measured at FLUXNET-station Hainich in 2007. (a) daily precipitation [mm], (b) daily air temperature [°C], (c) daily incoming radiation [photoactive photon flux density µmol/(m²s)].

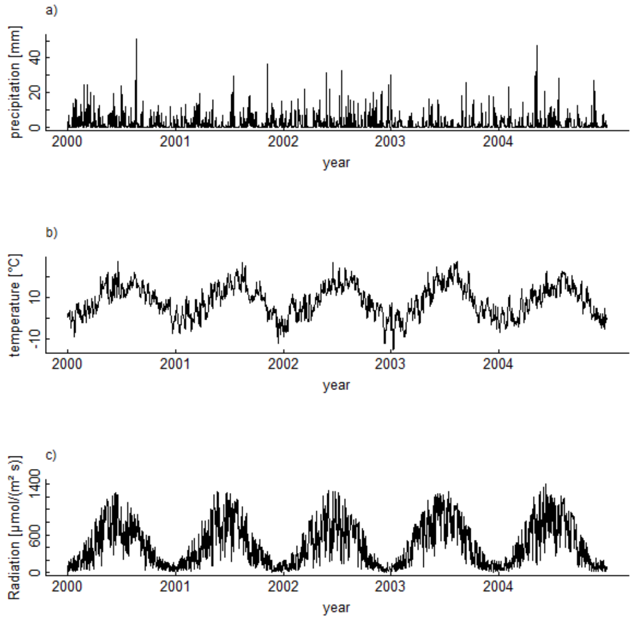

Figure A2: Overview of climate conditions used as input for the final productivity calculation. The climate data set was measured at FLUXNET-station Hainich from 2000 to 2004. (a) daily precipitation [mm], (b) daily air temperature [°C], (c) daily incoming radiation [photoactive photon flux density µmol/(m²s)].

A.2 Detailed description of generation of the forest patches.

The used stem size distributions are based on a Weibull-distribution (e.g.: Ryniker et al. 2006, Taubert et al. 2013). The peaks of the stem size distribution are set at a stem diameter of 5,15,25,35 and 45 cm whereby the 95% quintile are set at a stem diameter of 6,16,26,36 and 46 cm (figure A3). We selected this 15 stem size distribution to cover a broad range of forest structure in a systematic way and to limit calculation time. With this setting, we include 65% of all forest plots of the German forest inventory (35% are forest plots, which include at least on tree with a dbh larger than 50cm). However, larger maximal stem diameters are possible.

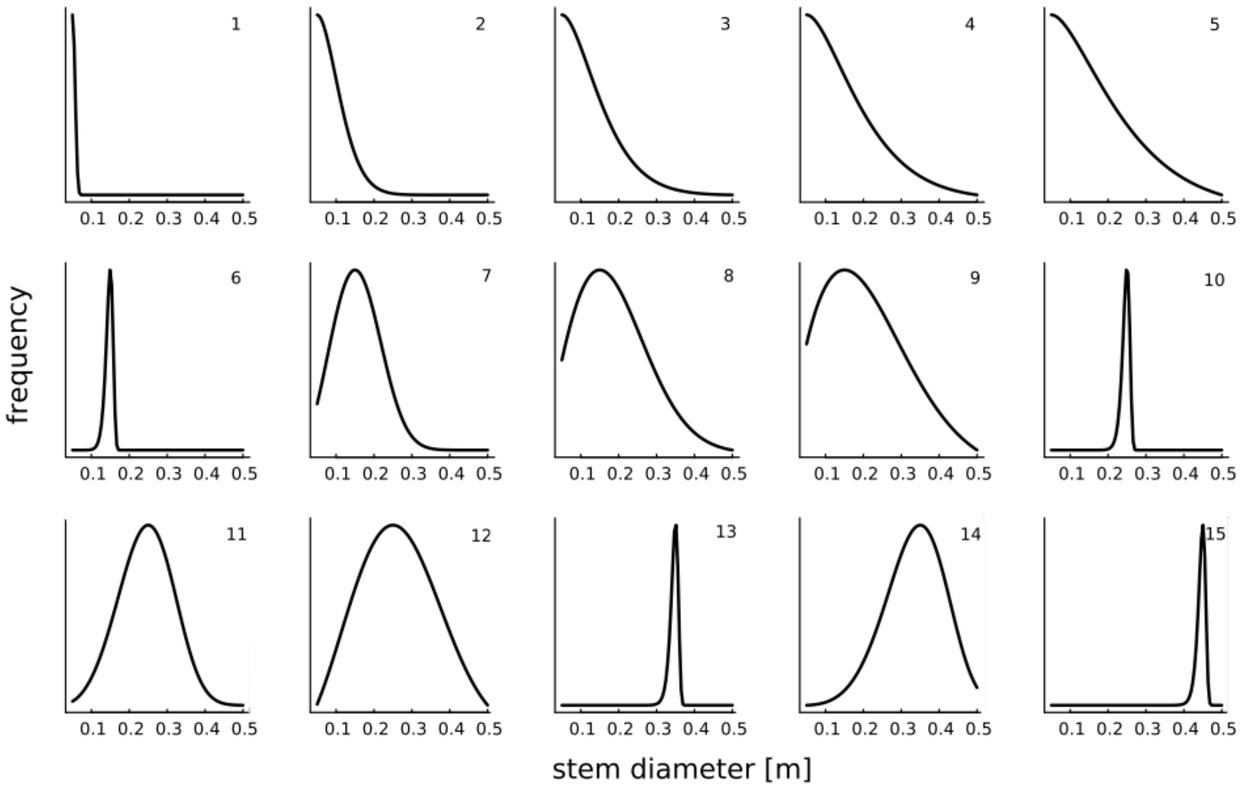

Figure A3: Overview of the different stem size distributions.

For every combination of species mixtures and stem size distributions we generate 100 patches of 400m² (= 4 hectare in total). For every hectare (25 patches) we execute step one and step two.

Step one: The input stem size distribution contains no information regarding the total number of stems. We therefore determine how many trees can be found per hectare by iteratively adding trees to reproduce the character of the stem size distribution as well as possible (rule 2). The only limitation in this step is the space occupied by the tree crowns (rule 1). Species identities of trees are chosen

randomly from the current species composition. Note that species differ in their crown allometries, which results in different stem numbers for different species composition, but the same stem size distribution. This step results in the maximal stem number per hectare of the current stem size distribution (see figure 1).

Step two: We start the placement of trees in the 25 patches of the stem size distribution resulting from step 1 with the largest tree (defined by the largest stem diameter) followed by the next smaller one. Every tree is assigned to one of the patches randomly. Before every tree placement we check

a) …if there is enough space in the patch for the tree crown. If not another patch is selected randomly. If no patch could take up the tree the total number of trees for the current hectare is reduced and the filling process of step two restarts.
b) …if the assigned species identity of the tree has a positive productivity under the current light and environmental conditions (figure 2, figure A4). If this is not the case another species identity (if available) is tested for positive productivity. If no species identity (of the current mixture) has a positive productivity, another patch is selected and the placement starts with the original species identity. If no patch could host the current tree the total number of trees in the hectare is reduced and the filling process of step two restarts.
c) … if the change in light conditions results in a negative productivity of any other tree in the patch. If this is the case, we check if a species identity with same stem diameter and positive productivity is available, which shades other trees less than the original one so that all trees have a positive productivity. If not all criteria are fulfilled another patch is selected. If no patch could host the tree the total number of trees for the current hectare is reduced and the filling process of step two restarts.

Note, if the tree number of the stem size distribution must be reduced, the reduction is executed in a way that keeps the shape of distribution at the hectare scale as good as possible.

The described procedure is implemented in R 3.2.2

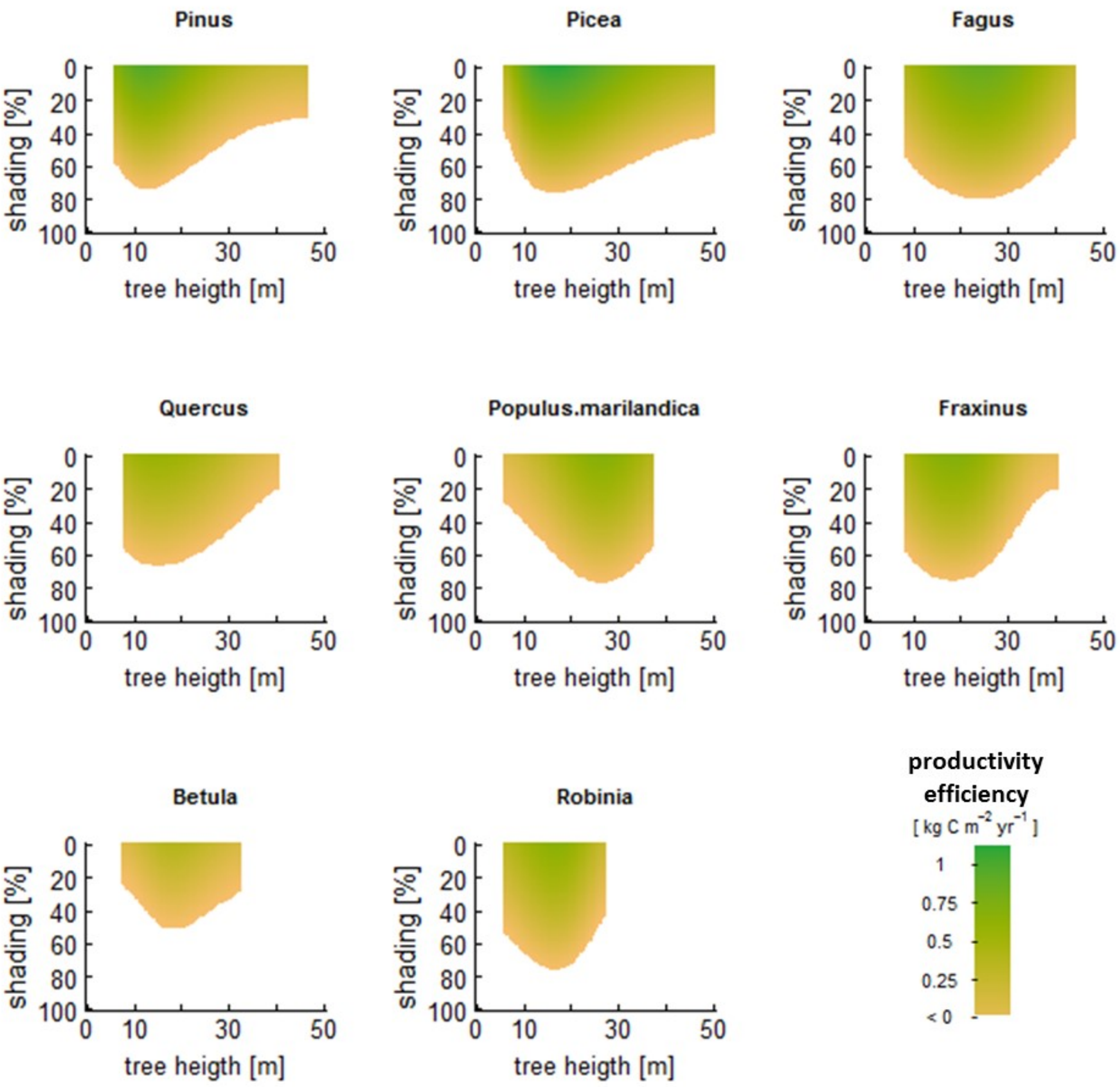

Figure A4: productivity efficiency (AWP_{tree} per unit crown area) of all eight species. Productivity depends on tree height and available light at the top of the tree under the given environmental conditions (Hainich 2007). Productivity efficiencies of tree heights with a dbh smaller than 5 cm and light-height combinations with negative productivity are not plotted (white area).

A.3 forest stand properties

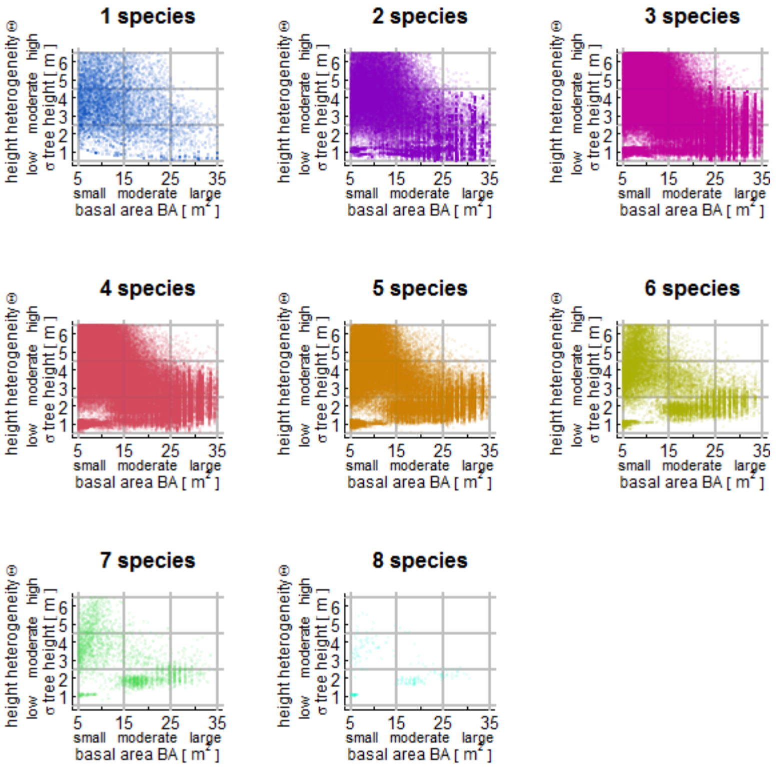

Figure A5: Overview of the all forest stands. Scatterplots between basal area and tree height heterogeneity, where each dot represents one forest stand. Grey lines separate the nine different structure classes of low, moderate and high tree height heterogeneity levels (0.5-2.5 m, 2.5-4.5 m and 4.5-6.5 m) and small, moderate and large basal areas (5-15 m², 15-25 m² and 25-35 m²).

A.4 Validation of AWP calculation.

We use eddy flux measurements to determine how well the forest factory method reproduces forest productivity under different climatic conditions (boreal and temperate). We also use German forest inventory II and III data to determine whether differences in forest structures and species mixture influence productivity. Note that the species-specific parameterization has already been tested successfully against the yield tables presented by Bohn et al. (2014).

For the validation with eddy flux measurements (Luysseart et al. 2007), we select sites with a homogenous forest structure, with trees of similar height within the footprint and with one dominant species (even-aged forests). For such sites, it is reasonable to assume that productivity per area is scale independent. From our dataset, we selected a forest stand from the forest factory model which hosts the identical species and which has the same LAI and forest height recorded for the flux site. The forest stand productivity was calculated from climate data measured at the sites. For temperate forests,

productivity was reproduced effectively (figure A5a). However, simulated and observed productivity were found to be less prevalent in the boreal zone.

To compare our AWP-estimation with the German forest inventory III (2012), we selected forest plots that (i) host only species considered in this study and (iii) are located on flat terrain (sloped at less than 15 %). As the inventory is based on variable radius sampling, we only consider plots with trees with a maximum dbh of 0.5 meters (which results in a maximal area of ~ 400 m² of the plots).

To construct the plots, we used the average height and dbh of each tree for inventories II and III. For the productivity calculations, we used Hainich station climate data (for 2000 to 2004) because plot-specific climate data were not available. We omitted stands presenting negative productivity (0.97% stands were removed). Simulated forest stand productivity levels correspond quite well with the observed stand productivity level (figure A6 b; figure A7 shows analysis with all values). Our slight underestimation of AWP (a = 0.85) may be a product of the assumed climate because the climate at the Hainich site is slightly colder than the average climate for Germany (the site is located at 430 m o.s.l). Such differences may also be attributable to specific tree allometries and local environmental conditions.

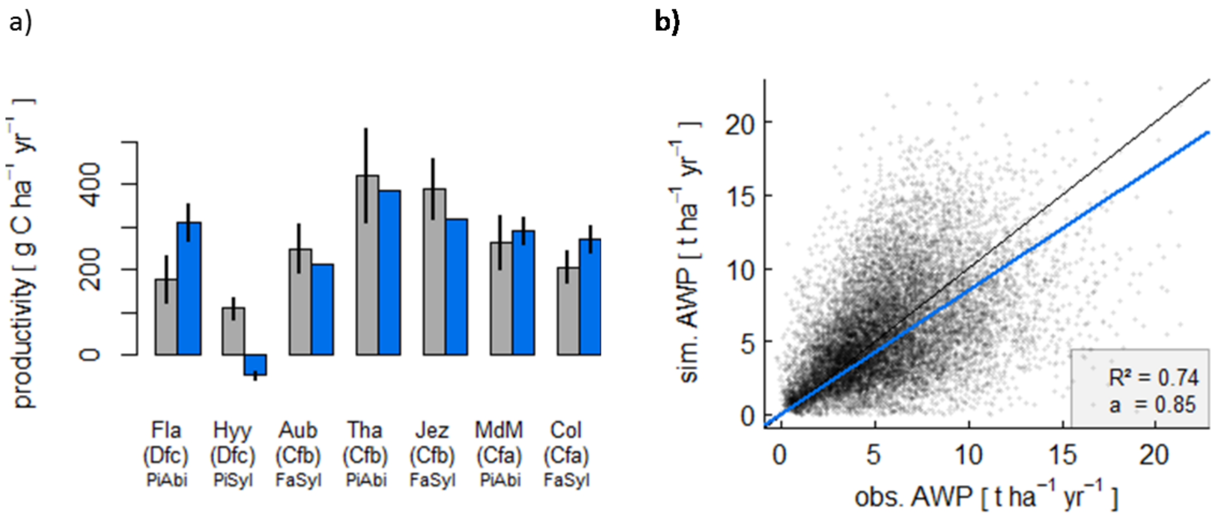

Figure A6: Validation for (a) eddy flux measured wood productivity (dark grey) and modelled AWP (blue). The following sites were selected: Flakaliden (Sweden), Hyytiala (Finland), Aubure (France), Tharand (Germany), Jezeri (Czech Republic), Monte di Mezzo and Collelongo (Italy). Dfc sites are located in a wet boreal climatic zone with cold summers, whereas Cfa and Cfb are located in a wet temperate climatic zone with hot (Cfa) and warm (Cfb) summers. Dominant species found in the sites are spruce (PiAbi), pine (PiSyl) and beech (FaSyl). b) Observed aboveground wood production for German forest inventory III

compared to simulated values. The black line represents the 1:1 line, and the blue line represents the linear fit between observed and simulated values using a linear model (a*x ; $R^2 = 0.74$; a =0.85).

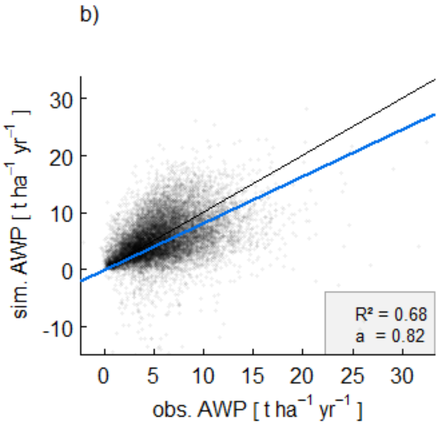

Figure A7: validation graphic including all forest plots. Every point represent compares the productivity of a forest plot of the German forest inventory with the simulated productivity of that plot. Points are transparent.

References

- Ryniker K, Bush J, Van Auken O. Structure of quercus gambelii communities in the Lincoln national forest, New Mexico, USA. *Forest Ecol. and Manag*. **2006**; 233: 69–77 (doi: 10.1016/j.foreco.2006.06.008)
- Taubert F, Hartig F, Dobner HJ, Huth A. On the challenge of fitting tree size distributions in ecology. *PLoS ONE*, **2013**; 8: e58036 (doi: 10.1371/journal.pone.0058036)
- Luyssaert S, Inglima I, Jung M, Richardson A, Reichstein M, Papale D, Piao SL, Schulze E-D, Wingate L, Matteucci G, et al. 2007, CO2 balance of boreal, temperate, and tropical forests derived from a global database. Glob. Change Biol. 13, 2509–2537 (doi: 10.1111/j.1365-2486.2007.01439.x)
