# Supplementary material for: The importance of forest structure to biodiversity–productivity relationships
Source: R Soc Open Sci. 2017 Jan 4;4(1):160521. doi: 10.1098/rsos.160521 (PMC5319316; doi:10.1098/rsos.160521)
Supplement: Appendix B: Additional information regarding results and discussion [file rsos160521supp2.pdf]

Appendix B

B.1 Impact of temperature and functional diversity on the results.

To explore the sensitivity of the results in figure 5 to changes in the temperature of the used climate time series, we reconduct the full analysis using a modified climate time series (we alter the temperature time series by 1.5°C, resulting in a mean annual temperature of 6.8°C and 9.8°C). In general, the observed pattern persists (figure B1, first & second rows). There is a slightly positive effect for forest stands with low height heterogeneity but a negative effect for forests with high height heterogeneity. Additionally, the variability of the productivity between the forest stands increases with increasing temperatures.

To analyse the effect of functional diversity on productivity (instead of richness), we calculate Rao´s Q (Rao 1982, Laliberte & Legendre 2010) using all of the physiological parameters that are related to the productivity calculation (n=12). However, the effect of functional diversity on productivity is negligible (figure B1). The variability in productivity did not decrease with increasing Rao´s Q as it did for species number.

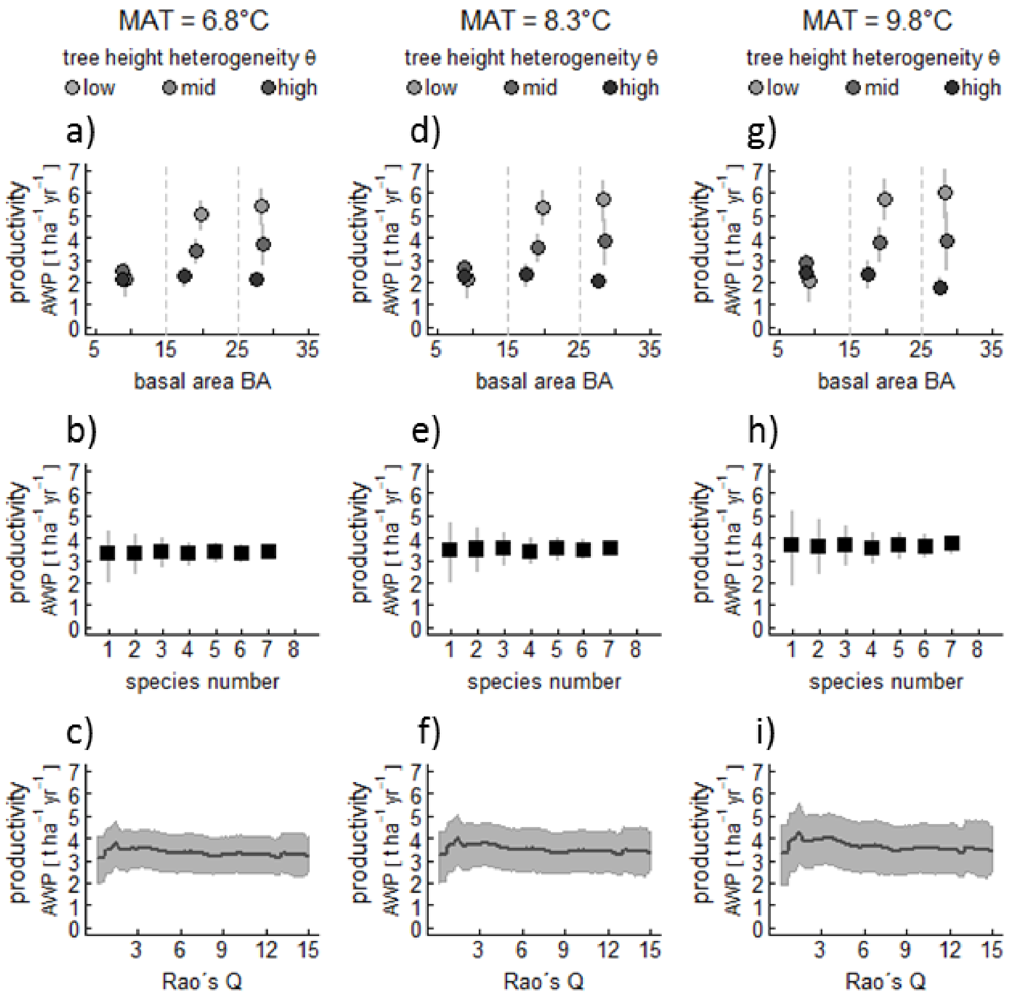

Figure B1: Sensitivity of forest stand productivity (above-ground wood production) against mean annual temperature (MAT). Left column based on simulation with a MAT of 6.8°C (figure a), b), c), middle column (figure d), e), f)) based on the measured data of Hainich and right column based on a simulation with a MAT of 9.8°C (figure g), h), i)). Mean productivity of the nine structure classes figure (a), d), g)): low, mid and high basal area BA (5-15 m² hectare^{-1}; 15-25 m² hectare^{-1}; 25-35 m² hectare^{-1}) and low mid and high tree height heterogeneity Θ (O.5-2.5 m; 2.5-4.5 m; 4.5-6.5 m); Mean productivity depending on species number (figure b), e), h) of forest stand. Mean productivity depending on Rao´s Q (figure c), f), i)). Grey bars indicate the interquartile range.

B.2 Analysis of the species richness within the German forest inventory

We analyse the relationship between diversity and productivity by using two different methods. First, we calculate the AWP_N for the plots of the German forest inventory based on $AWP_{mixsture}$, which consists only of beech, spruce or pine trees or one of their mixtures. This selection was made because other mixtures occur only in a small number of forest structure classes (in total we analysed 5,054 forest stands). Second, we calculate the mean productivity of all plots containing the same number of species (as conducted, for example, by Vilà et al 2007). With the second analysis, we find an increase in productivity of 10 % between one and two species mixtures, which corresponds to the findings of other studies (e.g., Vilà et al 2007). The calculated AWP_N instead shows no effect of diversity, which corresponds to the analysis of the forest factory (figure B1 and figure 5). When the mean productivity for all forested areas with a certain number of species was calculated, we found a 10 % increase in productivity between one and two species mixtures. This corresponds to the results of other inventories (e.g., Vilà et al 2007, Catalonian forest inventory). This positive relationship may be attributed to the fact that 71% of German forest inventory plots show height heterogeneity levels of less than 2.5 and basal areas of greater than 25 m². For these structure classes, our results show a positive diversity-productivity relationship (figure 6).

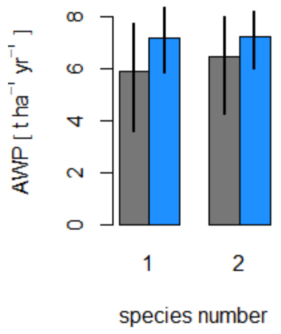

Figure B3: Mean productivity of forest stands (above-ground wood production) depending on species number for stands of the German forest inventory, which includes only beech, spruce and pine and their mixtures. Grey bars represent the mean productivity over all plots with the corresponding species number. Blue bars represent mean productivity (= $\mathbf{AWP_N}$ of the Manuscript), where we build the mean over all $\mathbf{AWP_{s,n}}$ while keeping the species number constant. Lines represent the interquartile range.

B.3 Forest stands with only one or two species

The relationship between forest structure and productivity (figure B1) can be analyzed for stands with only one species. Thereby the general pattern (productivity increases with increasing basal area and decreasing height heterogeneity) can be found in all mixtures (figure B4, B5). However, monocultures vary in their absolute productivity values (figure B4), but monocultures and AWP_N for all monocultures shows the general pattern quite well. In case of two species mixture with beech (figure B5) the differences of the productivity-structure-relationships between the mixtures are much lower and vanish almost completely for species mixtures with more than two species

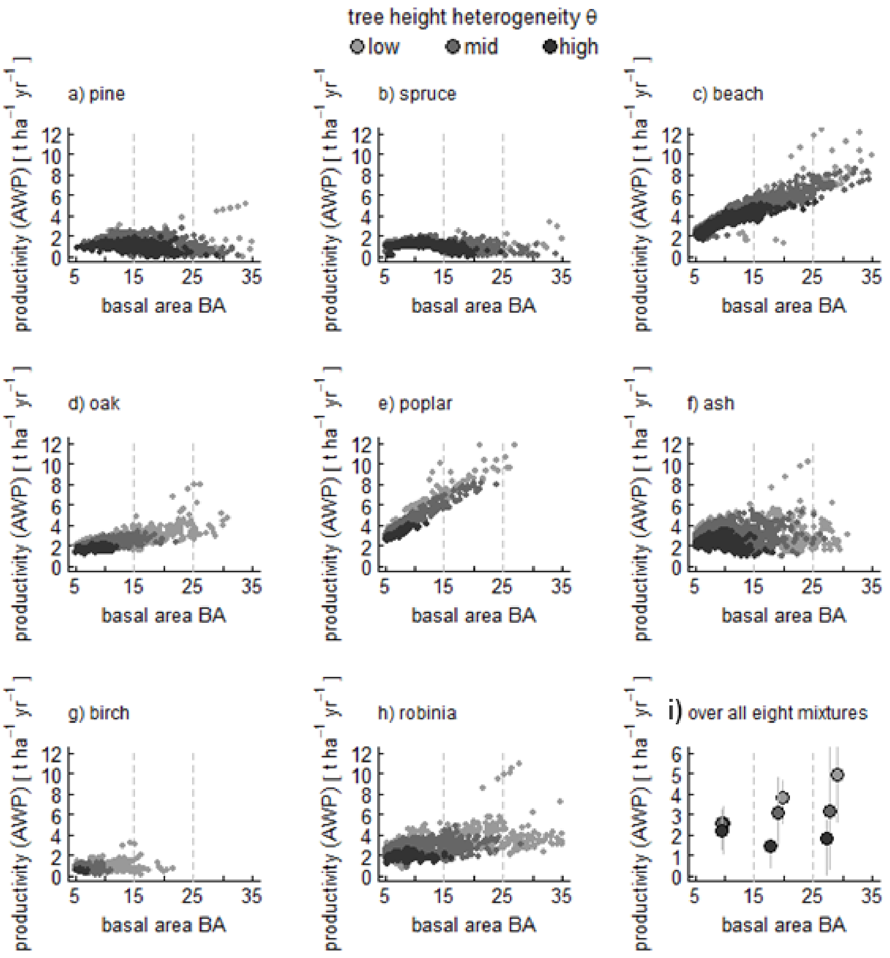

Figure B4: Analysis of the structure-productivity relationship of monocultures (a-h); every dot represent one forest stand. Darker greys indicate higher height heterogeneity classes. AWP_N values over all eight monocultures (i) with IQR as grey stripes.

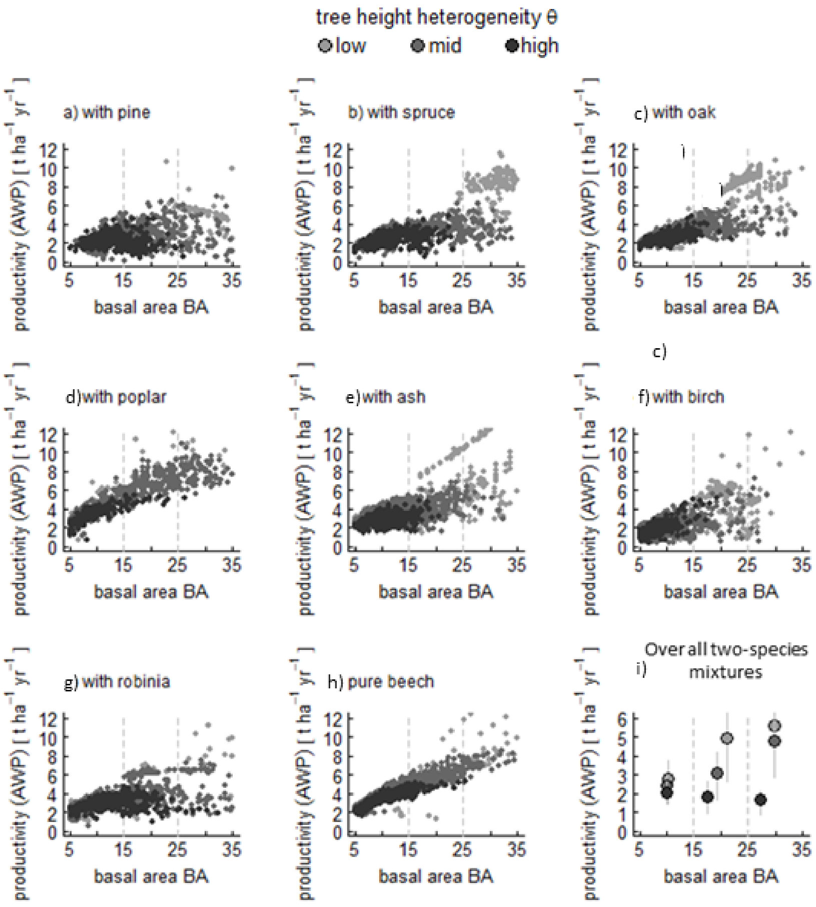

Figure B5: Analysis of the structure-productivity relationship of two-species-mixtures with beech (a-g) and beech monoculture (h); every dot represent one forest stand. Darker greys indicate higher height heterogeneity classes. (i) AWP_N over all seven two-species mixtures with IQR as grey stripes.

B.4 additive Partitioning analysis

Based on the concept of Loreau & Hector (2001) we perform additional partitioning analysis. As the forest factory does not include information about age we use as monocultures the average of those monocultures which show a similar forest structure. The structure indices (BA, Θ) are z-transformed so that both have a mean of 0 and a standard deviation of 1. We select the 10 nearest monocultures using Euclidian distance (95% of the structural distances between monocultures and the mixtures are below 0.22 in the z-transformed structure and the average distance is 0.08).

The overall analysis of the forest stands shows that both complementarity/ selection mechanisms have low potential to explain the variance of the forest productivity (figure B6). This finding does not change if we use relative abundances (in terms of biomass or basal area) for the calculation of the expected yield.

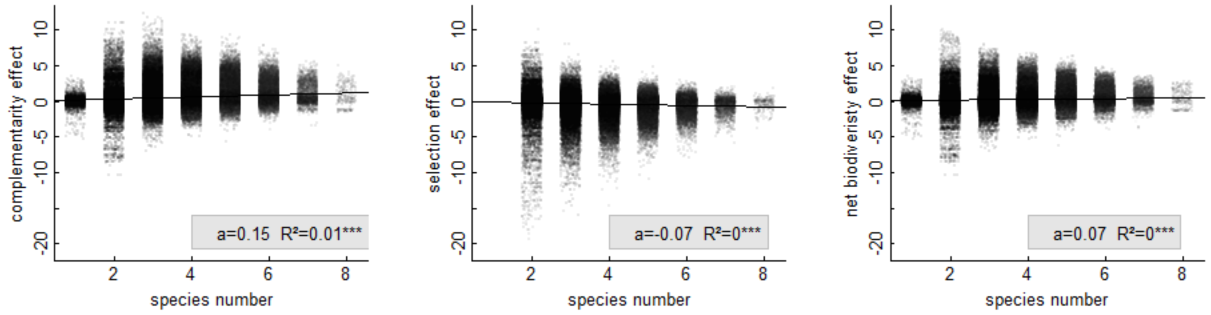

Figure B6: additive partitioning. a) complementarity; b) selection; c) net biodiversity effect. Line is a linear model with a as slope. Stars indicate significance of the model: *** indicates a p-value <0.001. Every dot represents one forest stand.

The analysis of the nine different forest structure classes shows also hardly any correlation between selection/complementarity and forest productivity.

B.5 example of the application of structure-optimality-mechanism

We analyze the relationship between diversity and the three indices of the structure-optimality-mechanism (figure B7) by calculating the coefficient of determination for all structure classes. The correlation between species number and optimal species distribution (Ω_{AWP}) or forest structure indices are on average much higher than the correlation found in the additive partitioning analysis (figure B8) and reach an R^2 of up to 0.25. Please note that a high correlation between species number and one index does not automatically result in a strong correlation of that index with the productivity. For instance, in the forest structure class with high basal areas and low tree height heterogeneity species number correlate quite well with structure indices (figure B9 a &b) but it correlates weak with Ω_{AWP} (figure B9 c)).However, Ω_{AWP} is the main driver of productivity in this structure class (figure B9 f).

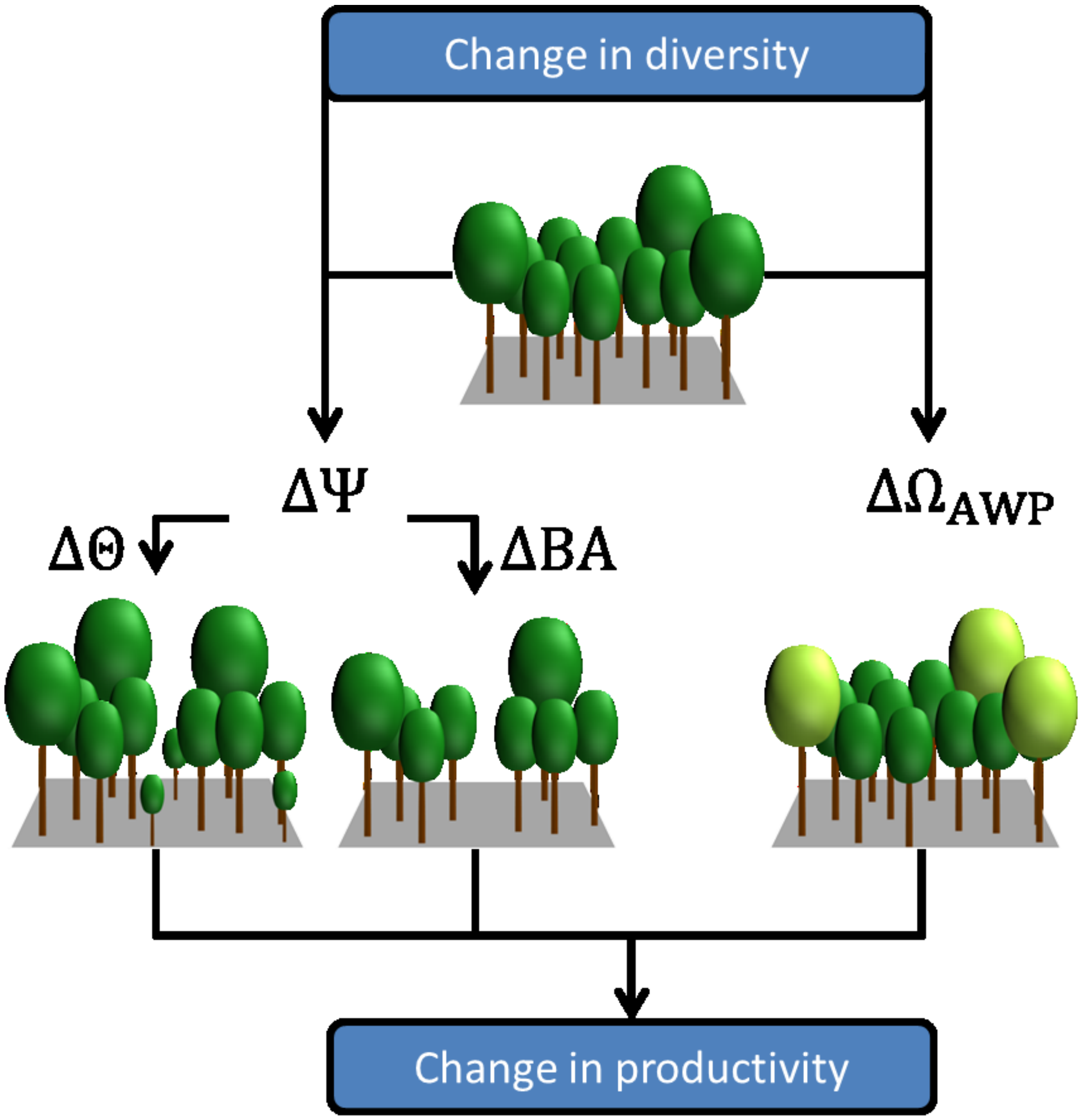

Figure B7: Concept of the structure-optimality-mechanism, which convert a change in diversity into a change of productivity. A change in tree diversity between two forests result in a change of forest structure $\boldsymbol{\Psi}$ and/or Ω_{AWP} . The change in forest structure splits into a change of tree height heterogeneity Θ and/or in a change of basal area.

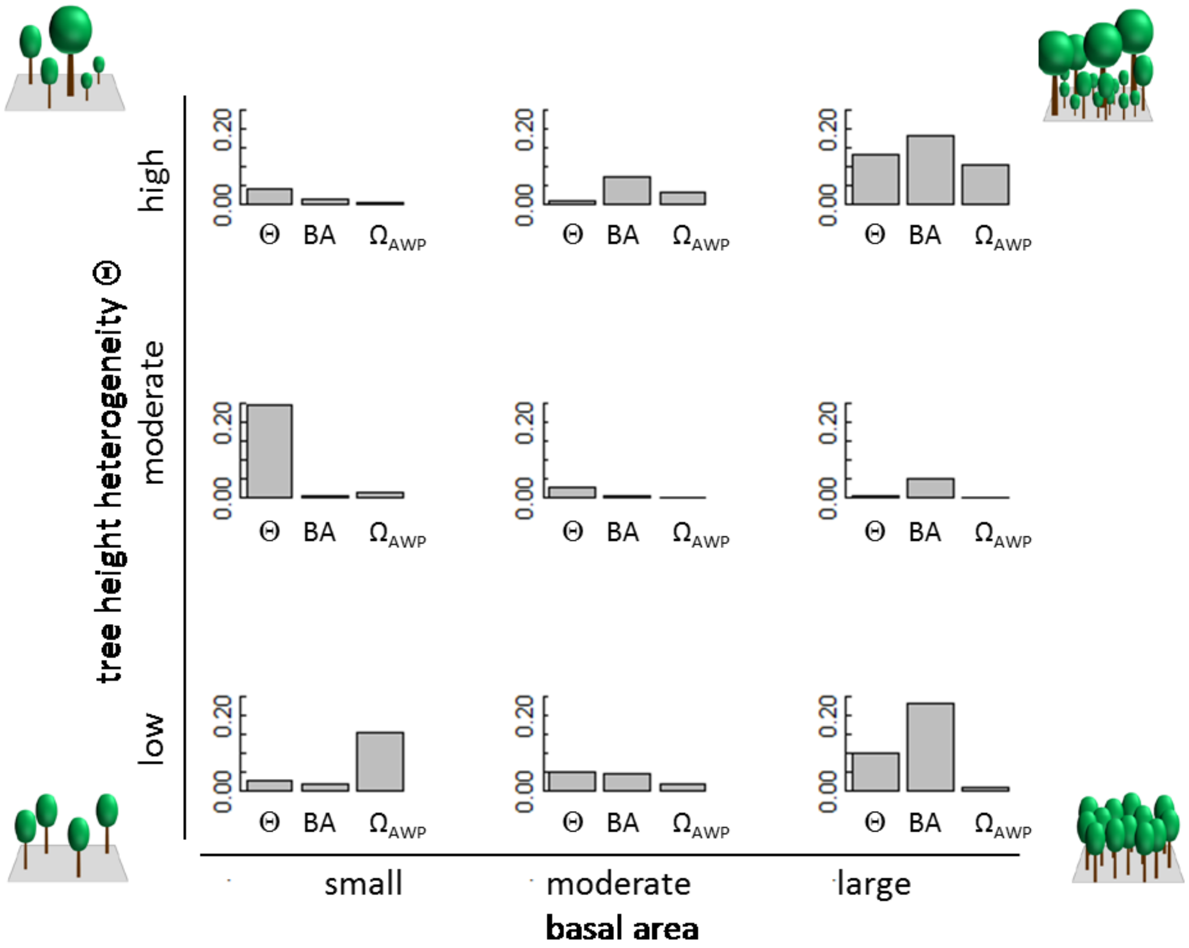

Figure B8: Coefficient of determination (R^2) between number of species and the indices of the structure-optimality-mechanism for the nine structure classes.

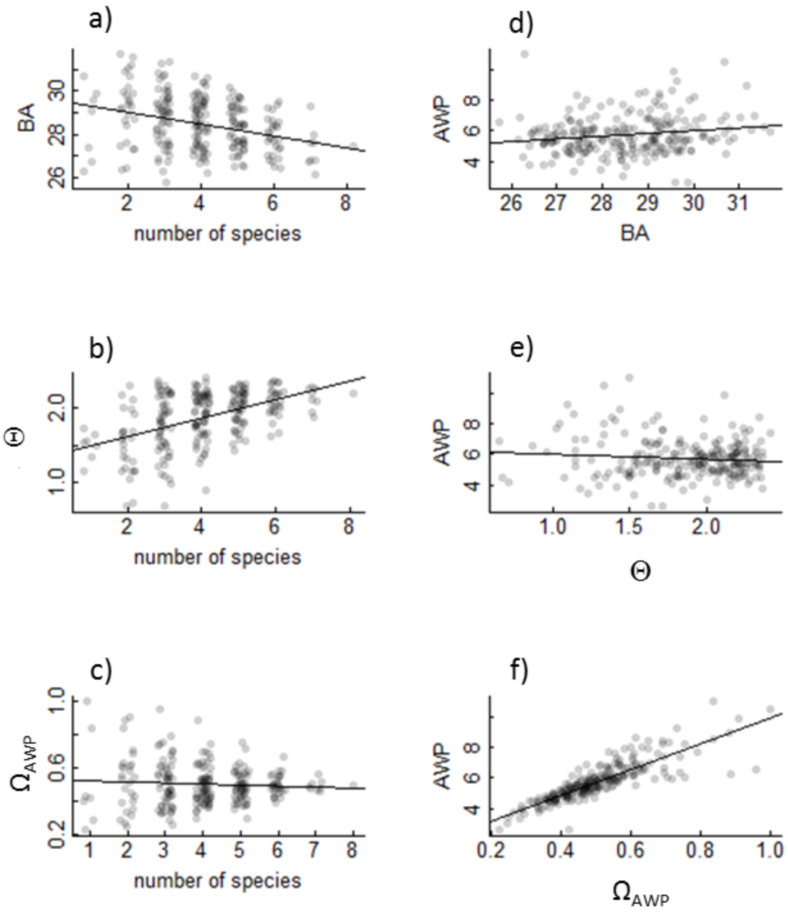

Figure B9: Relationship between number of species and forest structure indices (Θ and BA) as well as optimality (Ω_{AWP}) for forest stands with high basal area and low tree height heterogeneity (a, b, c). Relationship between forest structure indices (Θ and BA) and optimality (Ω_{AWP}) with forest productivity (AWP) (d, e, f). Every dot represents one forest stand. Black line shows a fitted linear model.

B.6 Analysis of forest stands with equal abundances of species.

To quantify, how strong unequal abundances of species due to rule 3 influence the results we repeat the analysis with a subsample of the forest factory data set. This data set includes only those forest

stands which have a functional evenness greater than 0.9 (Laliberte & Legendre 2010). Note that the calculation of functional evenness requires at least 3 different species. The analysis of the subsample shows a quite similar pattern as the analysis including all forest stands. Only forest stands with high basal area and high tree height heterogeneity show lower productivities.

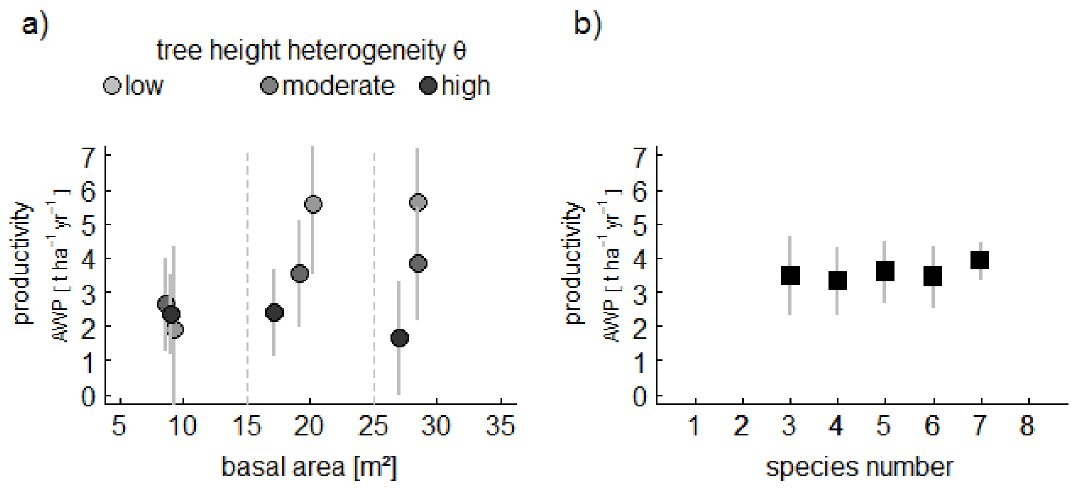

Figure B10: Analysis of mean productivity (aboveground wood production) of those forest stands, which show a functional evenness greater than 0.9. a) Mean productivity of the nine structure classes: small, moderate and large basal area BA (5-15 m² hectare^{-1}; 15-25 m² hectare^{-1}; 25-35 m² hectare^{-1}) and low, moderate, and high tree height heterogeneity ⍰ (O.5-2.5 m; 2.5-4.5 m; 4.5-6.5 m); b) Mean productivity depending on the numbers of species in a forest stand. Grey bars denote the mean standard deviation.

B.7 The influence of mean tree height and tree height heterogeneity on productivity

Different indices were developed to describe forest structure (Pommerening et al. 2002). We tested also the effect of mean tree height by replacing basal area in the analysis (figure B11). We observed similar patterns compared to the original analysis.

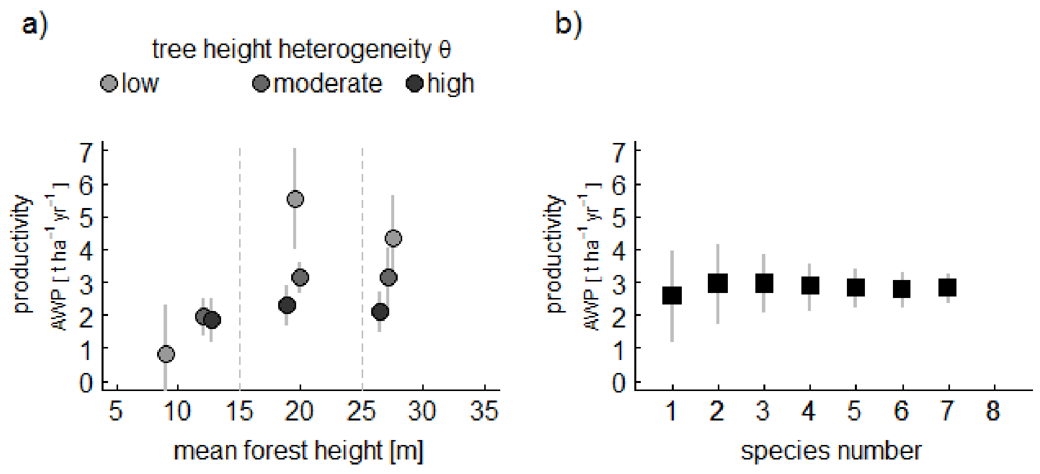

Figure B11: Analysis of mean productivity (aboveground wood production) of those forest stands. a) Mean productivity of the nine structure classes: small, moderate and large mean tree height (5-15 m; 15-25 m; 25-35 m) and low, moderate, and high tree height heterogeneity ϴ (O.5-2.5 m; 2.5-4.5 m; 4.5-6.5 m); b) Mean productivity depending on the numbers of species in a forest stand. Grey bars denote the mean standard deviation.

B.8 Diversity-productivity relationships covering only one forest structure class.

Beside the comparison between the forest stands of the forest factory with analysis of large data set, which based on forest inventories and cover several forest structure classes, subsamples of the forest factory can be compared with small datasets which belong only to one forest structure class.

Positive diversity-productivity relationship:

Many studies have analysed forest productivity in two or three species mixture experiments (e.g. Edgar et al. 2001, Chen et al 2003, Amoroso et al 2007, Pretzsch et al 2010). For instance, Edgar et al. 2001 analysed pure aspen stands and stands with admixtures of other species. The analysed forest stands are described by a top canopy height of ~ 25 m, a high basal area and a medium ϴ. For the analyzed forest stands an increase of basal area with diversity was found (basal area increase of 30%). Beside a positive structure mechanism (figure B7), the optimality-mechanism might also support the positive effect: In the monocultures larger aspen trees shade some smaller ones. In the mixture, smaller trees belong mostly to more shade-tolerant species and aspen only occur in the top layer which should result in an increase in Ω_{AWP}. Thus, the positive effect of diversity on productivity results from positive correlation between diversity and structure as well as Ω_{AWP}. This change in both forest properties (structure and Ω_{AWP}) is then responsible for the increase in productivity (figure B7). In other studies sometimes a separation over height of the species is described (e.g Pretsch et al. 2010) or a change in forest structure can be related to the observed productivity (e.g Amoroso et al 2007, Chen et al 2003).

Negative diversity-productivity relationship:

A decreasing relationship between diversity and productivity was found by Jacob et al. 2010 in the Hainich forest (Germany). They analysed nine forest plots which all show high basal areas, high tree height heterogeneityand cover an area of 50x50 meter. The plots contain only deciduous tree species (more than six) whereby the monocultures are dominated by beech (abundance = 96%). For the corresponding forest stands of the forest factory (same structure class only deciduous trees) (n =16) a negative relationship between Shannon-diversity and productivity can be observed which fits to the field

observations. Our analysis of the structure-optimality-mechanisms reveals a strong effect of Ω_{AWP} (R^2 = 0.91) and no effect of structure (R^2=0.01). Thus, the negative relationship can be explained by the fact that beech is the most productive species for all sizes of trees in such a forest (figure 4, area A). The low diverse forests in the study are dominated by beech resulting in the maximal productivity (high Ω_{AWP}). If beech trees are replaced by trees of other species (due to an increase in diversity) the productivity have to decrease. In this example diversity has a negative effect on Ω_{AWP}, while structural effects can be neglected (figure 7). The result is a negative diversity-productivity-relationship. This negative effect also occurs if we include also evergreen species (spruce and pine).

Literature

- Amoroso M.M. and Turnblom E.C. Comparing productivity of pure and mixed Douglas-fir and western hemlock plantations in the Pacific Northwest. *Can. J. For. Res.* **2006.** 36: 1484–1496
- Chen HYH, Klinka K. Aboveground productivity of western hemlock and western red cedar mixed-species stands in southern coastal British Colombia. *Forest Ecol. Manag*.**2003** 184: 55–64 (doi: 10.1016/S0378-1127(03)00148-8)
- Edgar CB, Burk TE. Productivity of aspen forests in northeastern Minnesota, U.S.A., as related to stand composition and canopy structure. *Can. J. Forest Res*. **2001** 31: 1019–1029 (doi:10.1139/x01-029
- Jacob M, Leuschner C, Thomas FM, Productivity of temperate broad-leaved forest stands differing in tree species diversity. *Ann. for. Sci.* **2010;** 67: 503 (doi: 10.1051/forest/2010005)
- Laliberte E. and Legendre P. A distance-based framework for measuring functional diversity from multiple traits. *Ecology.* **2010**; 299–305
- Pommerening, A. 2002, Approaches to quantifying forest structures. Forestry, 75, 305-324 (doi: 10.1093/forestry/75.3.305)
- Pretzsch H, Block J, Dieler J, Dong PH, Kohnle U, Nagel J, Spellmann H, Zingg A. Comparison between the productivity of pure and mixed stands of Norway spruce and European beech along an ecological gradient, *Ann. for. sci.* **2010**; 67: 712 (doi: 10.1051/forest/2010037)
- Rao, C. R. Diversity and dissimilarity coefficients—a unified approach. *Theoretical Population Biology.* **1982** 21:24–43.

- Vilà M. Vayreda J, Comas L, Ibáñez JJ, Mata T, Obón B. Species richness and wood production: a positive association in Mediterranean forests. *Ecol. Lett.* ***2007***; 10: 241–250 (doi: 10.1111/j.1461-0248.2007.01016.x)
