# Supplementary material for: The importance of forest structure to biodiversity–productivity relationships
Source: R Soc Open Sci. 2017 Jan 4;4(1):160521. doi: 10.1098/rsos.160521 (PMC5319316; doi:10.1098/rsos.160521)
Supplement: Appendix C: detailed Model describtion [file rsos160521supp3.pdf]

A General concept

FORMIND 3.0 is an individual-based, spatially explicit and process-based model designed for simulating species-rich vegetation communities. This document introduces only a specific version of FORMIND 3.0 (SVN-Built 1080) which simulates forest dynamics at central Europe. For a full model description of FORMIND 3.0 please go to www.formind.org. The full description shows the entire range of different model versions, which can be currently applied (i.e. the choices of different geometries of the vegetation, of the climatic zone or of various disturbance events).

In FORMIND 3.0 vegetation is simulated on an area of size A_{area}, which is a composite of regularly ordered, quadratic patches of size A_{patch} [m^2] uniquely described by their location within the area (Fig. 1). Individual trees grow within the patches, but do not have spatially explicit positions within a patch.

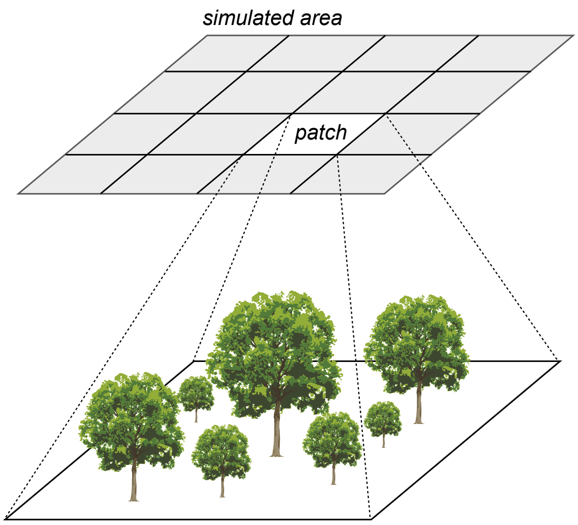

Figure 1: *Illustration of the simulated area and its composition of regularly ordered patches. Individual trees do not have spatially explicit positions within the patches. Only for an illustrative purpose, we show positioned trees on an exemplary patch.*

The trees change their size during the simulation according to a type-specific set of eco-physiological and morphological parameters used within the modelled processes. The modelled processes are simulated on different levels: (i) area-level, (ii) patch-level or (iii) on the level of a single tree .

Within each time step t_y, the following main processes are calculated:

- **Chapter C - Environment**
The patches of the simulation area are homogeneously concerning climatic input variables. Based on these input parameters, the environment of the trees is specified. For example, the radiation above canopy and day length are equal for all patches. The vertical attenuation of the incoming radiation (i.e. light climate) is calculated for each patch based on the vegetation, so that light intensity at different heights can differ between patches dependent on the number of trees shading each other. Reduced light availability for shaded individuals can result in a reduced gross photosynthesis. Limited soil water resources can also reduce the gross photosynthesis of an individual. In the same manner as the light climate, soil water contents can differ between patches during the simulation, although the initial soil water content and other soil properties (e.g. soil porosity) are equal for all patches. Differences in soil water content between patches are dependent on the number of trees per patch, which take up soil water resources. Further, type-specific effect of air temperature can also limit gross photosynthesis and affect respiration of an individual. All limitations are calculated in time steps of higher resolution than t_y.

- **Chapter D - Growth**
The growth of a single tree is determined by its gross productivity, respiration and type-specific morphological parameters. Respiration is calculated on the level of an individual. An increase in biomass per tree is modelled as the difference between gross photosynthesis and respiration. The allocation of the resulting biomass increase (including the increase of geometrical properties according to chapter B) act on the level of a tree .

The modelled processes, which are summarized within the above mentioned main processes, are scheduled in a serial way. For details on the modelled processes and their schedule see Fig. 2.

For the purpose of calculations within the processes of light climate and crowding mortality, the above-ground space is discretized into vertical height layers of constant width Δh. Table 1 shows general input parameters.

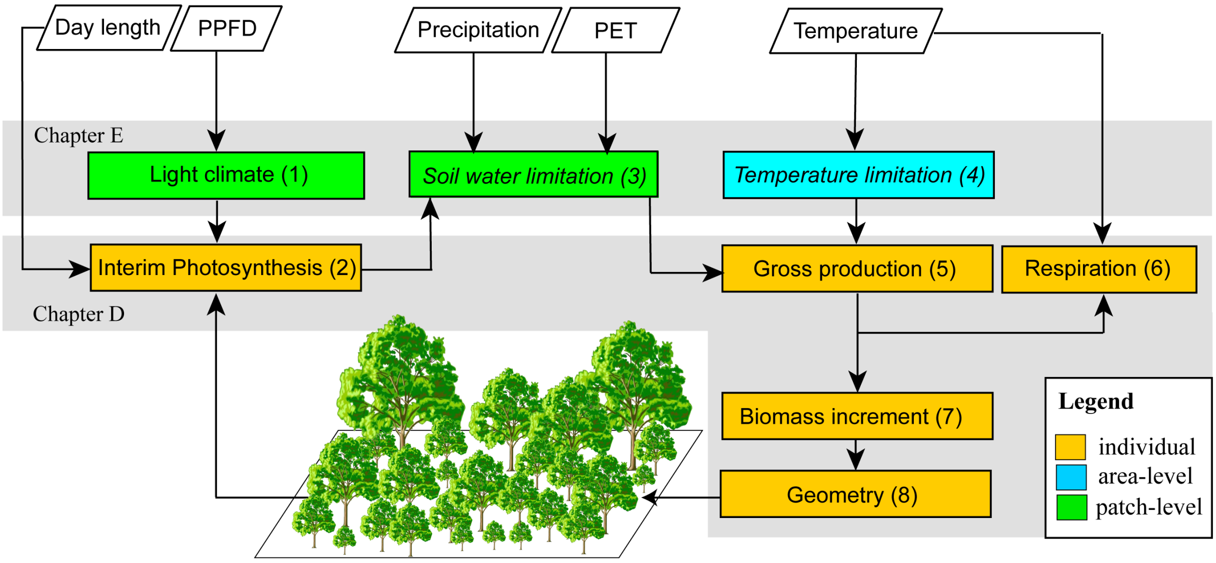

Figure 2: *Block diagram of the modelled processes. Different colours indicate the spatial scale on which each process is calculated (blue = area, green = patch, orange= individual). Italic written boxes show processes which are simulated with time steps of higher resolution than* t_y*. Numbers in brackets within each box show the serial order of their calculation within one time step* t_y*. Grey frames that underly these boxes group them according to the above mentioned main processes and their corresponding chapters. Rhombuses indicate climatic input parameters with the following abbreviations: PET – potential evapotranspiration, PPFD – photoactive photon flux density.*

Table 1: *General and technical parameters.*

Name	Symbol	Value	Unit
Simulation time	t_{end}	1	year
Time step	t_y	1	year
Simulation area	A_{area}	1	hectare
Patch area	A_{patch}	400	m^2
Number PFTs	$MaxGrp$	up to 8	-
Width of height layers	Δh	0.5	m

B Geometry

Although individual trees in real forests should not have necessarily identical shapes, we model each tree by a cylindrical stem and a cylindrical crown (Fig. 3). The geometry of an individual can be described completely by the following size characteristics: stem diameter (D), height (H), crown diameter (C_D), crown length (C_L) and crown projection area (C_A) as shown in Fig. 3.

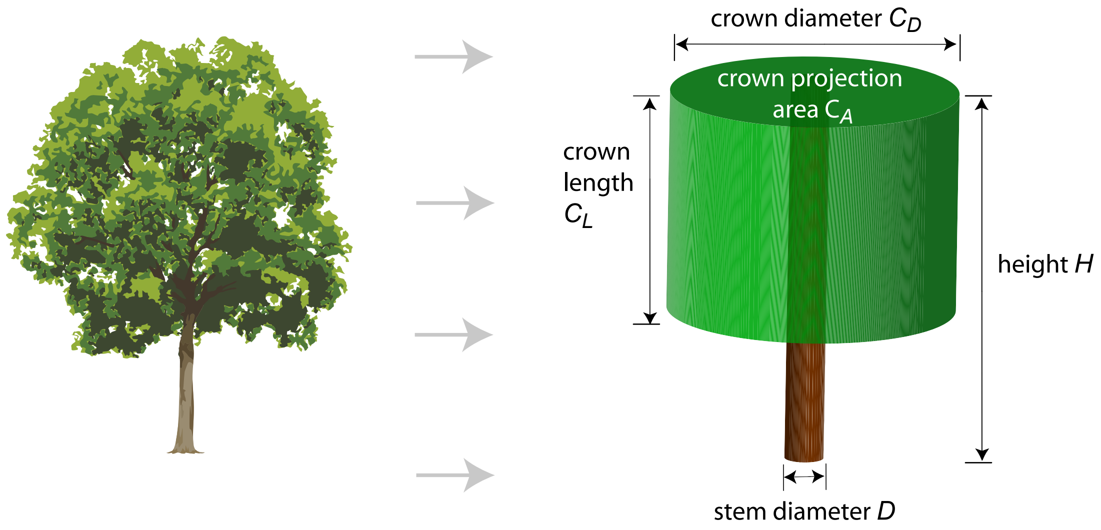

Figure 3: *Geometrical representation of a single tree . The following abbreviations describe size characteristics of the modelled tree geometry: D - stem diameter, H - height, C_D - crown diameter, C_L - crown length, C_A crown projection area.*

These size characteristics are functionally related to each other. In the following, we describe the functional relationships used. Parameters of the described relationships can vary between different tree types.

B.1 Height - Stem Diameter - Relationship

The height H [m] of a tree relates to its stem diameter D [cm] by:

$$H = \frac{D}{\frac{1}{h_0} + \frac{D}{h_1}}, \tag{1}$$

where h_0 and h_1 are type-specific parameters.

B.2 Crown length - Height - Relationship

The crown length C_L [m] of a tree is modelled as a fraction of its height H [m]:

$$C_L = (-\frac{c_{l0} \cdot H \cdot c_{l1}}{c_{l0} \cdot H + c_{l1}} + c_{l2}) \cdot H, \tag{2}$$

where c_{l0}, c_{l1} and c_{l2} are type-specific parameter.

B.3 Crown diameter - Stem diameter - Relationship

The second dimension of the cylindrical crown, i.e. the crown diameter C_D [m] of a tree relates to its stem diameter D [cm] by:

$$C_D = D \cdot (c_{d0} + c_{d1} \cdot exp\left(-c_{d2} \cdot D\right)), \tag{3}$$

where c_{d0}, c_{d1} and c_{d2} are type-specific parameters.

B.4 Crown area - Crown diameter - Relationship

The crown projection area C_A [m^2] of a tree is simply the ground area of the modelled cylindrical crown:

$$C_A = \frac{\pi}{4} \cdot C_D^2. \tag{4}$$

B.5 Aboveground biomass - Stem diameter - Relationship

The aboveground volume of a tree captures biomass (i.e. organic dry matter). Aboveground biomass B in [t_{ODM}] of a tree is modelled in relation to its stem diameter D [cm] by:

$$B = exp\left(b_0 \cdot (log(D) - b_2) \cdot \frac{2 \cdot b_1 + (log(D) - b_2)}{b_1 + (log(D) - b_2)}\right), \tag{5}$$

whereby b_0, b_1 and b_2 are type-specific parameters.

B.6 Leaf area index - Stem diameter - Relationship

In general, aboveground biomass is divided between woody biomass captured in the stem and green biomass captured in the crown leaves. Important for the photosynthetic production of a tree is the green biomass captured in crown leaves. As leaves absorb radiation for photosynthesis, the total amount of one-sided leaf area per unit of crown projection area (i.e. the individual's leaf area index) is of main interest. The leaf area index LAI [m^2/m^2] of a tree relates functionally to its stem diameter D [cm] by:

$$LAI = l_0 \cdot D^{l_1}, \tag{6}$$

whereby l_0 and l_1 are type-specific parameters.

All parameters mentioned above are listed in Tab.2.

Table 2: *Summary of the type-specific morphological parameters based on Schober [1995] yield class 1 (exeption: populus: only yield class 2 available)*

tree type	h_{max}	b_1	b_2	b_3	h_1	h_2	c_1	c_2	c_3
pinus	46	1.185	5.657	3.676	1.259	75.762	0.156	0.152	0.204
picea	50	1.029	3.204	3.717	1.326	101.33	0.128	0.102	0.089
fagus	43.7	1.202	5.727	3.475	1.916	61.036	0.155	0.125	0.066
quercus	40	1.151	5.187	3.586	1.879	45.341	0.173	0.054	0.066
populus	37	1.266	5.636	3.809	1.286	62.651	0.173	0.614	0.087
fraxinus	40	1.192	5.957	3.534	1.976	52.925	0.171	0.146	0.066
betula	32	1.091	6.394	3.671	1.711	51.488	0.207	1.760	0.277
robinia	27	1.217	9.175	3.586	1.400	45.315	0.161	0.493	0.120

B.7 Maximum Values

The trees cannot grow indefinitely in FORMIND 3.0 . Therefore, we introduce the following maximum values for a plausible geometry of a mature individual:

- maximum stem diameter D_{max} [m]
- maximum height H_{max} [m]
- maximum biomass B_{max} [t_{ODM}]

Either the maximum stem diameter or the maximum height is given as a type-specific input parameter. Those two maximum values, which are not predefined, are then derived using the functional relationships mentioned in section B.1 and section B.5. The maximum values are used in section D.

C Competition and environmental limitations

C.1 Light climate

A single tree on a patch receives full incoming radiation. An increasing number of individual trees of differing heights on a patch results in shading within the canopy. Higher trees partly intercept radiation, which is not available for smaller individuals. Thus, with decreasing height from the canopy down to the ground, radiation is increasingly attenuated. We call this vertical distribution of light availability within a patch 'light climate'.

To calculate the light availability in different heights within the canopy, the vertical discretization of the above-ground space is used (i.e. height layers of constant width Δh). For each patch and height layer, the leaf area accumulated by all trees on the patch is calculated. Each tree contributes parts of its crown leaf area to those height layers, which are occupied by its crown (i.e. height layers from l_{min} to l_{max}). These limits are determined by the individual's crown length C_L and its height H:

$$l_{max} = \left\lfloor \frac{H}{\Delta h} \right\rfloor \tag{7}$$

$$l_{min} = \left\lfloor \frac{H - C_L}{\Delta h} \right\rfloor . \tag{8}$$

The number of height layers a tree is occupying by its crown ($\#_{layer}$) can then be calculated by:

$$\#_{layer} = l_{max} - l_{min}. \tag{9}$$

For those height layers between l_{min} and l_{max}, an individual's leaf area contributes equally to each layer i:

$$\bar{L}_i = \frac{LAI \cdot C_A}{\#_{layer}}, \tag{10}$$

whereby $\bar{L}_i$ [m^2] represents the contribution of an tree 's leaf area to the layer i, LAI [-] is the leaf area index of the tree (see section B.6) and C_A [m^2] is crown projection area of the tree 's crown. The multiplication of LAI by C_A results in the leaf area in [m^2] of an single tree .

Summing up all contributions of the trees ' leaf area per patch to their respective occupied height layers and relative to the patch area, results in the patch-based leaf area index $\hat{L}_i$ [-] per layer i:

$$\hat{L}_i = \frac{1}{A_{patch}} \sum_{\substack{all\ individuals \\ with\ l_{min} \leq i \leq l_{max}}} \bar{L}_i, \tag{11}$$

where $\bar{L}_i$ [m^2] represents the leaf area contribution of an tree to the height layer i and A_{patch} [m^2] denotes the area of a patch.

Using this information, the radiation each tree is able to intercept can be determined. Light attenuation through the canopy is calculated using the approach of [Monsi and Saeki, 1953]. The incoming radiation I_{ind} on top of a tree (i.e. on top of the height layer l_{max} the tree is reaching) is calculated by:

$$I_{ind} = I_0 \cdot exp\left(-k \cdot \sum_{i>l_{max}} \hat{L}_i\right), \tag{12}$$

where the sum in the exponent accumulates the patch-based leaf area indices of all height layers above the individual's height. The parameter k denotes the light extinction coefficient [-] of a tree , I_0 [μmol (photons)/m^2 s] is the daily radiation above canopy averaged from sunrise to sunset during the vegetation period and $\hat{L}_i$ [-] represents the patch-based leaf area index of height layer i.

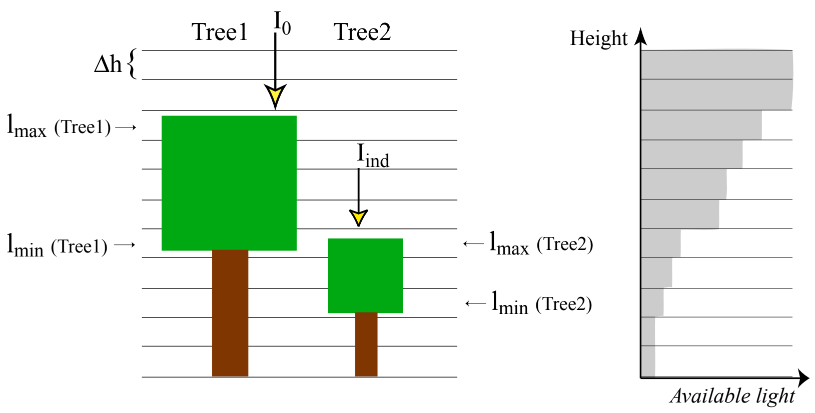

Figure 4: *Illustration of the light climate on the example of two single trees . The limits of each crown are shown by l_{min} (Tree1), l_{max}(Tree1), l_{min}(Tree2) and l_{max}(Tree2). The vertically discretized aboveground space into height layers of width Δh [m] is coloured differently according to the available radiation. The lighter the colour is, the more attenuated the radiation is, which results from the absorption by higher individuals' leaves. On the right hand side the decrease of available light from the canopy to the floor is illustrated by the grey polygon. Thereby, attenuation is greatest in the height layer both trees occupy by their crowns (i.e. layer l_{min}(Tree1) and l_{max}(Tree2)).*

By determining the available radiation for each single tree , competition for light between trees is considered.

Table 3: *tree type specific leaf area index (LAI) measurements of Breuer et al. [2003]. (n) is number of used measurements. Robinia is estimated as mean of quercus and populus because of same light value by Ellenberg.*

tree type	LAI m^2/m^2 (n)	sd
pinus	3.6 (2)	1.0
picea	7.7 (4	2.1
fagus	6.1 (6)	0.9
quercus	5.4 (4)	0.7
populus	4.6 (7)	1.6
fraxinus	5.0 (1)	-
betula	5.3 (1)	-
robinia	5.0 (0)	-

C.2 Water cycle and soil water limitation

Individual trees take up soil water resources to fulfill the requirements for their gross productivity. Instead of modelling the roots of the trees , we determine an individual's uptake of soil water based on its demand and on the total available soil water.

Firstly, the soil water content Θ_{soil} is computed preliminary on an hourly basis using a differential equation, which quantifies preliminary hourly changes in the soil water content per patch depending on precipitation PR, interception IN and run-off RO (Fig. 5, cf. [Kumagai et al., 2004]):

$$\frac{d\Theta_{soil}}{dt} = PR(t) - IN(t) - RO(t). \tag{13}$$

The resulting soil water content represents the total available soil water before soil water uptake by individuals. Uptake of soil water resources by trees is modelled equal to their transpiration and subtracted from Θ_{soil} later within the timestep (see eqn. 24).

The **interception** IN [mm/h] is calculated dependent on the total leaf area index per patch (i.e. $\sum_i \hat{L}_i$ in [-], cf. [Liang et al., 1994]):

$$IN(t) = min(K_L \cdot \left(\sum_i \hat{L}_i\right), PR(t)), \tag{14}$$

where K_L [mm/h] is the interception constant and PR [mm/h] denotes the precipitation.

On the ground surface of a patch, we consider two different run-offs: surface run-off and subsurface run-off:

$$RO(t) = RO_{\rightarrow}(t) + RO_{\downarrow}(t), \tag{15}$$

where **surface run-off** $RO_{\rightarrow}$ [mm/h] is defined in the following way:

$$RO_{\rightarrow} = max(0, \Theta_{soil}(t) + PR(t) - IN(t) - POR) \tag{16}$$

with POR [mm/h] denoting the soil porosity (i.e. defined as the maximum water intake of the soil per patch).

For the calculation of the **subsurface run-off** $RO_{\downarrow}$ due to gravitation, we use the Brooks-Corey relation (cf. [Liang et al., 1994]):

$$RO_{\downarrow} = K_s \cdot \left(\frac{\Theta_{soil}(t) - \Theta_{res}}{POR - \Theta_{res}}\right)^{\frac{2}{\lambda}+3}, \tag{17}$$

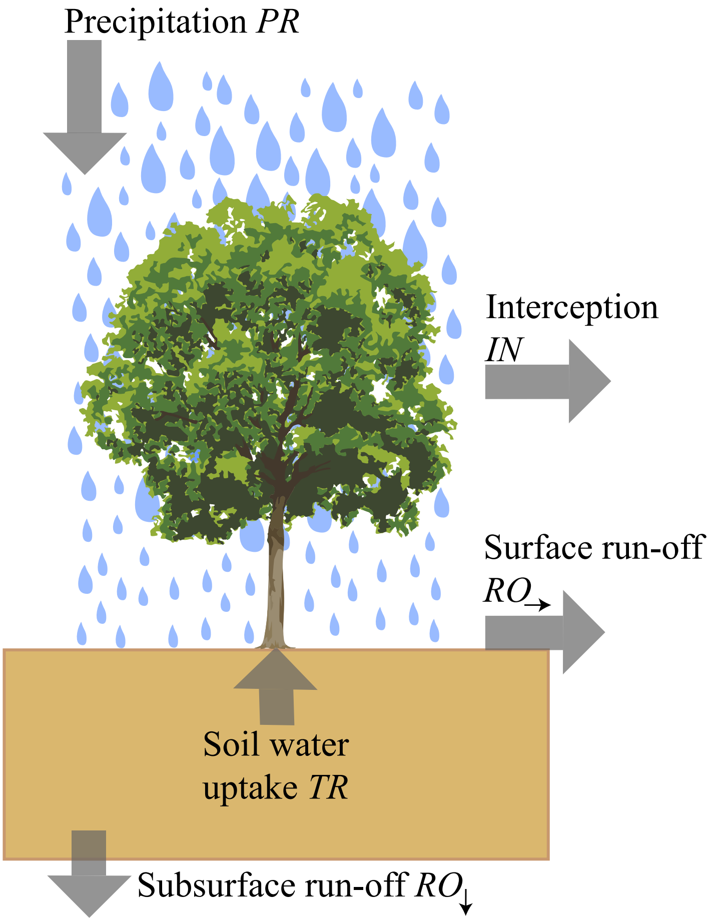

Figure 5: *Illustration of the water cycle on the example of a single tree .*

where K_s [mm/h] is the fully saturated conductivity, Θ_{res} [mm/h] the residual water content, and λ [-] the pore size distribution index.

The preliminary soil water content Θ_{soil} represents the soil water content, which is available for the individuals' uptake or transpiration. To calculate the **transpiration** TR [mm/h] of all trees per patch, we use the water-use-efficiency concept (cf. [Lambers et al., 2008]):

$$TR = \frac{1}{A_{patch}} \sum_{all\ trees} \frac{GPP}{WUE}, \tag{18}$$

whereby GPP in [g_{ODM}/h] denotes the hourly gross primary production of an individual on the patch (see section D). Please note, that we simulate GPP per time step t_y. To calculate GPP on an hourly basis, we divide GPP [g_{ODM}/t_y] by the number of hours within the time step t_y. The constant type-specific value WUE in [g_{ODM}/kg_{H_2O}] represents the water-use-efficiency parameter and A_{patch} [m^2] the area of a patch.

The resulting transpiration TR may be limited in three ways calculated in a serial way:

PET limitation Transpiration can be limited by the potential evapotranspiration PET [mm/h] and the interception IN [mm/h] (calculated by eqn. 14):

$$TR = \begin{cases} TR(t) & ,TR(t) \leq PET(t) - IN(t) \\ PET(t) - IN(t) & ,TR(t) > PET(t) - IN(t) \end{cases}. \tag{19}$$

Soil water limitation Transpiration can be limited by the preliminary soil water content Θ_{soil} [mm/h] (calculated by eqn. 13) and the permanent wilting point Θ_{pwp} [mm/h]:

$$TR = \begin{cases} TR(t) & ,\Theta_{soil}(t) - TR(t) \geq \Theta_{pwp} \\ \Theta_{soil}(t) - \Theta_{pwp} & ,\Theta_{soil}(t) - TR(t) < \Theta_{pwp} \\ 0 & ,\Theta_{soil}(t) \leq \Theta_{pwp} \end{cases}. \tag{20}$$

Competition for water Competition between trees can limit the transpiration in the following way:

$$TR = \varphi_W \cdot TR(t), \tag{21}$$

where φ_W [-] represents a reduction factor ranging between 0 and 1.

The reduction factor φ_W is calculated using the approach of [Granier et al., 1999], which is based on the preliminary soil water content (calculated by eqn. 13):

$$\varphi_W = \begin{cases} 0 & ,\Theta_{soil}(t) \leq \Theta_{pwp} \\ \frac{\Theta_{soil}(t)-\Theta_{pwp}}{\Theta_{msw}-\Theta_{pwp}} & ,\Theta_{pwp} < \Theta_{soil}(t) < \Theta_{msw} \\ 1 & ,\Theta_{soil}(t) \leq \Theta_{msw} \end{cases}, \tag{22}$$

Table 4: *water circle relevant parameters*

parameter	unit	value	reference
WUE	$g\ odm\ kg^{-1} H_2O^{-1}$	6.0	Larcher [2001]
Θ_{pwp}	$V\%$	19.4	Maidment [1993]
F_c	$V\%$	31.0	Maidment [1993]
k_L	$mm\ d^{-1}$	0.2	Dickinson [1984]
por	$V\%$	46.3	Maidment [1993]
k_s	$m\ s^{-1}$	3.66 10^{-6}	Maidment [1993]
Θ_r	$V\%$	2.7	Maidment [1993]

where Θ_{pwp} is the permanent wilting point in $[V\%]$ and Θ_{msw} is the minimum soil water content in $[V\%]$. For the purpose of the calculation of eqn. 22 only, Θ_{soil} needs to be converted from [mm/h] to [V%]. Thereby, the soil is modelled down to a constant depth [m] defined prior to the start of the simulation.

The value of Θ_{msw} is determined according to [Granier et al., 1999] by:

$$\Theta_{msw} = \Theta_{pwp} + 0.4(\Theta_{fc} - \Theta_{pwp}) \tag{23}$$

whereby Θ_{fc} denotes the field capacity in $[V\%]$.

The soil water content in the next day step is then calculated by the difference between the preliminary soil water content (calculated by eqn. 13) and the (eventually limited) transpiration TR:

$$\frac{d\Theta_{soil}}{dt} = \Theta_{soil}(t) – TR(t). \tag{24}$$

C.3 Temperature

The gross primary production GPP $[t_{ODM}/t_y]$ of a tree (see section D) may be influenced by phenology (esp. in the temperate zone) and air temperature. Respiration for maintenance purposes of an individual (see section D) may also be affected by air temperature. The influence on both - gross productivity and respiration, is modelled using limitation factor, by which they are simply multiplied (see section D). In the following, we describe the calculations of these limitation factors:

Phenology

Individual trees only produce gross primary production GPP during their photosynthetic active period. In the **temperate zone**, we distinguish between broad-leaf and needle-leaf trees . Only deciduous broad-leaf trees have two phenology phases: (i) a dormant phase during winter and (ii) a photosynthetic active period of φ_{act} [days] after bud-burst until fall (i.e. the vegetation period).

The date of bud-burst is reached, if the temperature sum (daily mean air temperatures $> 5°$) since 1 January is higher than a critical temperature T_{crit}:

$$T_{crit} = -68 + 638\ e^{-0.01 \cdot n}, \tag{25}$$

where n is the number of days per time step t_y with an air temperature below 5° since 1 November of the previous year. This algorithm is based on the global distribution of leaf onset dates estimated from remote sensing data [Botta et al., 2000]. The photosynthetic active period stops if the 10-day moving average of daily mean air temperatures falls below $9°C$ [Sato et al., 2007].

In contrast to the broad-leaf trees, the photosynthetic active period φ_{act} of needle-leaf trees amounts a complete year of 365 days (without any dormant phase).

In the **tropical zone**, we assume for all individuals irrespective of their type a complete photosynthetic active period with $\varphi_{act} = 365$ days.

Temperature limitation of gross productivity

The gross primary production of a tree can be reduced due to unfavorable air temperatures. A corresponding limitation factor φ_T is calculated by:

$$\varphi_T = \frac{1}{n} \sum_{1}^{n} \varphi_{T,l} \cdot \varphi_{T,h}, \tag{26}$$

where n is the number of days per time step t_y and the values $\varphi_{T,l}$ and $\varphi_{T,h}$ are the daily inhibition factors for low and high air temperatures [Gutiérrez and Huth, 2012; Haxeltine and Prentice, 1996].

The **inhibition factor for low air temperatures** $\varphi_{T,l}$ $[°C]$ is calculated by:

$$\varphi_{T,l} = \left(1 + e^{k_0 \cdot k_1 - T}\right)^{-1}, \tag{27}$$

where T $[°C]$ is the daily mean air temperature and k_0 and k_1 are type-specific parameters.

These parameters k_0 and k_1 are calculated by:

$$k_0 = \frac{2\ ln(0.01/0.99)}{T_{CO_2,l} - T_{cold}} \tag{28}$$

$$k_1 = 0.5\ (T_{CO_2,l} + T_{cold}) \tag{29}$$

where $T_{CO_2,l}$ $[°C]$ and T_{cold} $[°C]$ are type-specific parameters representing the lower temperature limit for CO_2 assimilation and the monthly mean air temperature of the coldest month an individual can cope with, respectively.

Similarly, the **inhibition factor for high air temperatures** $\varphi_{T,h}$ in $°C$ is calculated by:

$$\varphi_{T,h} = 1 - 0.01 \cdot e^{k_2\ (T - T_{hot})} \tag{30}$$

where k_2 is a type-specific parameter, T $[°C]$ is the daily mean temperature and T_{hot} $[°C]$ is the type-specific mean temperature of the hottest month an individual can occur.

The parameter k_2 is calculated as:

$$k_2 = \frac{ln(0.99/0.01)}{T_{CO_2,h} - T_{hot}}, \tag{31}$$

whereby $T_{CO_2,h}$ $[°C]$ and T_{hot} $[°C]$ are type-specific parameters representing the higher temperature limit for CO_2 assimilation and the monthly mean air temperature of the warmest month an individual can cope with, respectively.

Temperature limitation of maintenance respiration

Maintenance respiration is assumed to change exponentially with air temperature represented by the limitation factor κ_T [Prentice et al., 1993]:

$$\kappa_T = \frac{1}{n} \sum_{1}^{n} Q_{10}^{\left(\frac{T - T_{ref}}{10}\right)}, \tag{32}$$

where n is the number of days per time step t_y, T [$°C$] is the daily mean air temperature, Q_{10} [-] and T_{ref} [$°C$] are constant parameters, irrespective of type. T_{ref} represents the reference temperature, at which maintenance respiration is not influenced. Air temperatures below T_{ref} result in a decrease of maintenance respiration ($\kappa_T < 1$) and those above T_{ref} in an increase of maintenance respiration ($\kappa_T > 1$).

Table 5: *Warmest and coldest month where the tree species can cope with using distribution maps and the climate diagrams of J.Müller [1996].*

tree type	T_{hot} $°C$	T_{cold} $°C$	T_{ref} $°C$	Q_{10}
pinus	26.55	-2.33	10.47	2.3
picea	26.55	-9.9	10.47	2.3
fagus	22.00	-3.2	10.47	2.3
quercus	23.5	-3.9	10.47	2.3
populus	27.0	-6.90	10.47	2.3
fraxinus	25.55	-6.61	10.47	2.3
betula	21.5	-9.9	10.47	2.3
robinia	24.5	-9.1	10.47	2.3

D Growth of a tree

D.1 Interim photosynthesis

Based on the incoming irradiance on top of a tree I_{ind} (see section C), organic dry matter is produced via gross photosynthesis. In this section the interim photosynthesis is calculated without reduction due to limited soil water availability nor temperature effects.

The interim gross photosynthesis P_{ind} of an individual is modelled using the approach of [Thornley and Johnson, 1990]. It is based on the single-leaf photosynthesis modelled by a Michaelis-Menten function – a typical saturation function describing the relation between the radiation I_{leaf} available on top of a leaf and its gross photosynthetic rate P_{leaf}:

$$P_{leaf}(I_{leaf}) = \frac{\alpha \cdot I_{leaf} \cdot p_{max}}{\alpha \cdot I_{leaf} + p_{max}}, \tag{33}$$

where α is the quantum efficiency, also known as the initial slope of the type-specific light response curve, I_{leaf} is the incoming irradiance on top of the surface of a single leaf within the individual's crown and p_{max} is the maximum leaf gross photosynthetic rate.

To obtain the incoming irradiance on top of the surface of a single leaf I_{leaf}, the available irradiance I_{ind} on top of the entire individual has to be modified:

$$I_{leaf}(L) = \frac{k}{1-m}\, I_{ind} \cdot e^{-k \cdot L}, \tag{34}$$

where k [-] is the type-specific light extinction coefficient, m [-] represents the transmission coefficient and I_{ind} denotes the available incoming irradiance on top of a tree .

The first part $\frac{k}{1-m}\, I_{ind}$ in eqn. (34) is correcting the incoming irradiance in order to obtain those parts, which can be absorbed by a leaf. The second part $e^{-k \cdot L}$ in eqn. (34) accounts for self-shading within the individual's crown. As the leaves of an individual are assumed to be homogeneously distributed within its crown, some leaves will be shaded by higher ones within the crown. Thereby, $L = 0$ represents the top of the individual and $L = LAI$ represents the bottom of the individual's crown with LAI being its leaf area index (see section B).

To obtain the interim gross photosynthetic rate of a tree per year P_{ind}, the single-leaf photosynthesis of eqn. (33) is integrated over the individual's leaf area index LAI (see section B):

$$P_{ind} = \int_0^{LAI} P_{leaf}(I_{leaf}(L))dL. \tag{35}$$

The integration results in the interim photosynthesis of an tree per year [Thornley and Johnson, 1990]:

$$P_{ind} = \frac{p_{max}}{k} \cdot ln \frac{\alpha\ k\ I_{ind} + p_{max}(1-m)}{\alpha\ k\ I_{ind}\ e^{-k \cdot LAI} + p_{max}(1-m)}. \tag{36}$$

To convert the interim photosynthesis P_{ind} from $[\mu mol_{CO_2}/m^2 s]$ to $[t_{ODM}/y]$, P_{ind} has to be multiplied by the individual's crown area C_A (see section B), the type-specific photosynthetic active period φ_{act} and finally a conversion factor c_{odm}:

$$P_{ind} \cdot C_A \cdot 60 \cdot 60 \cdot l_{day} \cdot \varphi_{act} \cdot \varphi_{odm}, \tag{37}$$

where the multiplication by $60 \cdot 60$ accounts for the conversion from seconds to hours. The factor l_{day} [h] represents the mean day length during the vegetation period φ_{act} [d] (see section C). The conversion factor $\varphi_{odm} = 0.63 \cdot 44 \cdot 10^{-12}$ includes the molar mass of CO_2, the conversion from g to t and the conversion from CO_2 to organic dry mass ODM [Larcher, 2001].

D.2 Gross primary production

The gross primary production GPP of a tree is calculated from the interim photosynthesis P_{ind} $[t_{ODM}/y]$ (see section D.1):

$$GPP = P_{ind}\ \varphi_T\ \varphi_W, \tag{38}$$

where φ_W denotes the reduction factor accounting for limited soil water and φ_T represents the limitation factor of air temperature effect. Both factors range between 0 and 1 and thus, only reducing GPP in times of unfavorable conditions (see section C).

Table 6: *tree type specific photosynthetic parameters based on data of Sonntag [1998] (a). The rest is interpolated*

tree type	p_{max} $^{\mu mol_{CO_2}}/_{m^2 s}$	α $^{\mu mol_{CO_2}}/_{\mu mol_{photons}}$
pinus	18.82 a	0.0364 a
picea	14.1 a	0.0402 a
fagus	13.14 a	0.0644 a
quercus	16.87	0.0368
populus	14.69	0.0385
fraxinus	13.44	0.0471
betula	18.81	0.0364
robinia	14.1	0.0402

Table 7: general parameter and constants of forest

name	value	unit
mean global irradiance day length m	0.1	-
k	0.7	-

D.3 Biomass increment of a tree

Gross primary production GPP of eqn. (38) is first used for the maintenance of the already existing aboveground biomass of an tree . Costs for maintenance are modelled as biomass losses in terms of maintenance respiration R_m $[t_{ODM}/y]$. The remaining productivity $(GPP - R_m)$ is then available for growth of new aboveground biomass. Costs for the production of new structural tissue are modelled also as biomass losses in terms of growth respiration. This results in the net productivity ΔB [Dislich et al., 2009]:

$$\Delta B = (1 - r_g)\ (GPP - R_m), \tag{39}$$

where r_g [-] represents a constant parameter describing the fraction of $(GPP - R_m)$ attributed to growth respiration. In contrast, maintenance respiration R_m is modelled proportionally to the already existing aboveground biomass of a tree (see section D.4).

D.4 Maintenance respiration

The maintenance respiration R_m of a tree is calculated inversely by rearranging eqn. (39):

$$R_m = GPP - \frac{\Delta B}{1 - r_g}. \tag{40}$$

Maintenance respiration R_m is further modelled proportional to the already existing aboveground biomass B $[t_{ODM}]$ of an individual:

$$R_m = \kappa_T \cdot r_m \cdot B, \tag{41}$$

where r_m denotes the maintenance respiration rate [$^1/_y$] and κ_T represents a limitation factor dependent on air temperature (see section C).

Combining equation (40) with equation (41) and arranging in terms of the respiration rate r_m results in:

$$r_m = \frac{1}{B \cdot \kappa_T} \cdot \left(GPP - \frac{\Delta B}{1 - R_g} \right). \tag{42}$$

In this approach, the maintenance respiration rate r_m is calculated including those climatic conditions, which were observed during the field measurements of stem diameter

increments. The correspondence of environmental factors (see section C) to these climatic conditions during the observations is indicated by $\check{()}$.

$$r_m = \frac{1}{B} \cdot \left(GPP(\check{I_{ind}}, \check{\varphi_{act}}, \check{\varphi_T}, \check{\varphi_W}) - \frac{B(D + g(D)) - B}{(1 - R_g)} \right), \tag{43}$$

where this equation can be obtained by substituting in eqn. (42) (i) κ_T by 1, (ii) GPP by the gross productivity under the climate during observations $GPP(\check{I_{ind}}, \check{\varphi_{act}}, \check{\varphi_T}, \check{\varphi_W})$ and (iii) ΔB by the biomass increment derived from the maximum stem diameter increment using the individual's geometry $D + g(D)$ (see section B). See section D.5 for different modelling approaches of the *diameter growth curve* $g(D)$.

This approach is proposed when climate data are available at the time field data on stem diameter increments were measured. In general, diameter increments are determined based on the difference of stem diameter measurements between two dates. For this time period climate data would be needed on which the limitation factors $\check{I_{ind}}$, $\check{\varphi_{act}}$, $\check{\varphi_T}$ and $\check{\varphi_W}$ of eqn. (43) can be calculated as described in section C.

Table 8: *tree type specific carbon balance parameters*

tree type	ψ^*	I_r^*	$\varphi_T^* * \varphi_W^*$	d_1	d_2	d_3
pinus	1.00	503.79	0.9998	1.83 10^{-3}	3.216	1.253
picea			0.9975	5.67 10^{-3}	0.820	1.064
fagus	0.4839	761.23	0.9985	4.70 10^{-3}	1.252	1.39
quercus			0.9992	7.06 10^{-3}	0.703	1.184
populus			0.9999	14.32 10^{-3}	1.396	1.220
fraxinus			0.9997	2.04 10^{-3}	3.651	1.512
betula			0.9981	3.74 10^{-3}	1.445	1.445
robinia			0.9995	3.12 10^{-3}	3.393	1.120

D.5 Diameter growth curve

In the field, diameter increments can be determined by calculating the differences between two measurements of the stem diameter per tree (at two distinct observation dates). The increments are then usually plotted with the measured stem diameter of the first observation date to get an impression of how much a tree of stem diameter D is able to increase (see Fig. 6 for an example).

Such point clouds as illustrated in Fig. 6 can be described by functional relationships. Please note, that you have to adjust the increments according to a time step of 1 year.

That means, if there is a period of e.g. 5 years between both observation dates of stem diameter measurements, you would have to correct the increments with respect to the smaller time scale.

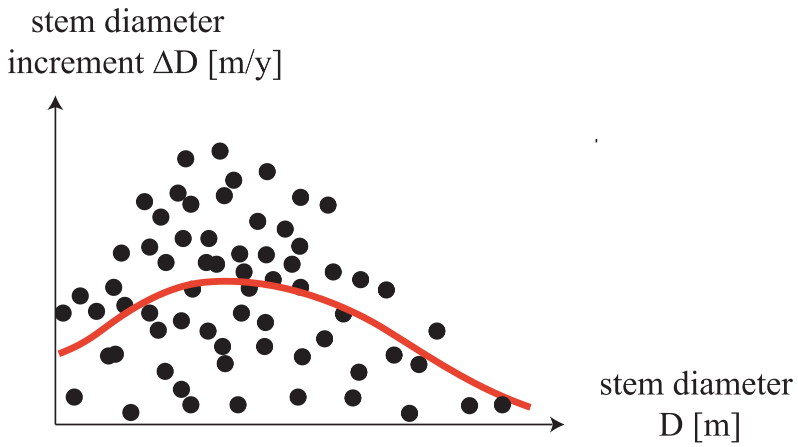

Figure 6: *Illustration of a diameter growth curve. Points represent illustrative measurements. The solid line represents a fitted growth function.*

For this approach, the coefficients of the corresponding growth function $g(D)$ are input parameter already known prior to the start of the simulation.

Weibull approach

The growth function $g(D)$ is described by a Weibull function of:

$$g(D) = a_0 \cdot a_1 \cdot a_2 \cdot (a_1 \cdot D)^{a_2 - 1} \cdot e^{-(a_1 \cdot D)^{a_2}}, \tag{44}$$

where a_0, a_1 and a_2 are the type-specific coefficients.
Please note, when determining the type-specific coefficients prior to the start of the simulation, that the curve represents growth under full resource availability. That means, not all measurements should be fitted, but only the maximum diameter increments (see Fischer, 2010 p. 55 for an example).

Table 9: *Summary of the type-specific growth and respiration parameters for all species. References:* [1] *calibrated* [2] *Ryan, 1991*

tree type	a_0	a_1	a_2	r_g
pinus	0.00183	3.216	1.253	0.25
picea	0.00567	0.82	1.064	0.25
fagus	0.0047	1.252	1.39	0.25
quercus	0.00706	0.703	1.184	0.25
populus	0.01432	1.396	1.220	0.25
fraxinus	0.00204	3.651	1.512	0.25
betula	0.00374	1.445	1.145	0.25
robinia	0.00312	3.393	1.12	0.25

E Input parameter and variables

Table 10: *General input parameter of the simulation.*

Symbol	Description	Unit
A_{area}	Simulation area	ha
A_{patch}	Patch area	m^2
$\#_{patches}$	Number of patches per simulation area	
t_y	time step	yr

Table 11: *Geometrical input parameter.*

Symbol	Description	Unit
h_0, h_1, h_2	Height-Stem diameter-Relationship	-
c_{l0}, c_{l1}, c_{l2}	Crown length-Height-Relationship	-
c_{d0}, c_{d1}, c_{d2}, c_{d3}	Crown diameter-Stem diameter-Relationship	-
ρ	Wood density	t_{ODM}/m^3
σ	Ratio of total aboveground biomass to stem biomass	-
f	form factor	-
f_0, f_1, f_2	Form factor-Stem diameter-Relationship	-
b_0, b_1, b_2	Biomass-Stem diameter-Relationship	-
l_0, l_1	LAI-Stem diameter-Relationship	-
D_{max}	Maximum stem diameter	m
H_{max}	Maximum height	m
B_{max}	Maximum biomass	t_{ODM}

Table 12: *Recruitment and establishment input parameter.*

Symbol	Description	Unit
N_{seed}	Global in-growth rate	$^1/_{\text{ha yr}}$
N_{init}	Initial seed number in seed pool	$^1/_{\text{patch}}$
D_{rep}	Minimum stem diameter of a recruiting mother tree	m
f_{disp}	Dispersal kernel	-
$dist$	Average dispersal distance	m
σ	Ratio of total aboveground biomass to stem biomass	-
I_{seed}	Percentage of incoming radiation at floor required for germination	%
M_{pool}	Seed pool mortality rate	$^1/_{\text{yr}}$
max_{dens}	Maximum number of germinated seedlings	$^1/_{\text{patch}}$
D_{min}	Stem diameter of a germinated seedling	m

Table 13: *Mortality input parameter.*

Symbol	Description	Unit
M_B	Basic mortality rate	$^1/_{\text{yr}}$
m_{d0}, m_{d1}	Mortality rate dependent on stem diameter	-
m_{i0}, m_{i1}, m_{i2}	Mortality rate dependent on stem diameter increment	-
N_M	Min. number of individuals at which stochastic dying is performed	$^1/_{\text{cohort}}$
D_M	Max. stem diameter below which stochastic dying is performed	m
t_{meadow}	Time ...	yr
t_{regrow}	Time ...	yr

Table 14: Light climate and photosynthesis input parameter and variables.

Symbol	Description	Unit
Δh	Width of layers of aboveground vertical space discretization	m
$\#_{layer}$	Number of layer of aboveground vertical space discretization	
I_0	Incoming irradiance on top of canopy	$\mu mol_{photon}/m^2\ s$
k	Light extinction coefficient	-
α	Initial slope of light response curve	$\mu mol_{CO_2}/\mu mol_{photon}$
p_{max}	Maximum leaf gross photosynthetic rate	$\mu mol_{CO_2}/m^2 s$
m	Transmission coefficient	-
l_{day}	Day length	h
φ_{ODM}	Conversion factor	$t_{ODM}/\mu mol_{CO_2}$

Table 15: Water module input parameter and variables.

Symbol	Description	Unit
PR	Precipitation	mm/h
K_L	Interception constant	mm/h
POR	Soil porosity	mm/h
K_s	Fully saturated conductivity	mm/h
Θ_{res}	Residual soil water content	mm/h
λ	Pore size distribution index	-
WUE	Water-use-efficiency	t_{ODM}/kg_{H_2O}
PET	Potential evapotranspiration	mm/h
Θ_{soil}^{init}	Initial soil water content at start of simulation	V%
Θ_{pwp}	Permanent wilting point	V%
Θ_{fc}	Field capacity	V%
Θ_{msw}	Minimum soil water content	V%

Table 16: *Temperature input parameter and variables.*

Symbol	Description	Unit
T	Air temperature	$°C$
n	Number of days per time step t_y	$1/t_y$
T_{crit}	Critical temperature for bud-burst	$°C$
k_0, k_1, k_2	Parameter of inhibition factors	-
$T_{CO_2,l}$, $T_{CO_2,h}$	temperature limits of CO_2 assimilation	$°C$
T_{hot}, T_{cold}	monthly mean temperature of warmest and coldest month an individual can cope with	$°C$
T_{ref}	Reference temperature	$°C$
Q_{10}	Base of Q10 function	-

Table 17: *Respiration input parameter and variables.*

Symbol	Description	Unit
R_g	Growth respiration factor	-
$g(D)$	Maximum stem diameter increment (growth) function	-
a_0, a_1, a_2, a_3	Coefficients of the growth function $g(D)$	-
x_i, $i = 1, ..., 8$	Auxillary variables	-
ΔD_{max}	Maximum measured stem diameter increment	m/y
$D_{\Delta D_{max}}$	Stem diameter at which maximum increment is measured	% of D_{max}
$\Delta D_{D_{min}}$	Max. measured stem diameter increment for diameter D_{min}	% of ΔD_{max}
$\Delta D_{D_{max}}$	Max. measured stem diameter increment for diameter D_{max}	% of ΔD_{max}
$\check{I_{ind}}$	Reference irradiance of parameterization climate	$\mu mol_{photon}/m^2 s$
$\check{\varphi_{act}}$	Reference vegetation period of parameterization climate	d
$\check{\varphi_T}$	Reference temperature limitation factor of photosynthesis of parameterization climate	-

F State variables

Table 18: *Geometrical state variables.*

Symbol	Description	Unit
D	Stem diameter at breast height	m
H	Height	m
C_D	Crown diameter	m
C_L	Crown length	m
C_A	Crown projection area	m^2
B	Aboveground biomass	t_{ODM}
LAI	Leaf area index	-
ΔB	Biomass increment per time step	t_{ODM}
ΔD	Diameter increment per time step	m

Table 19: *Recruitment and establishment state variables.*

Symbol	Description	Unit
N_{pool}	Seed pool (i.e number of seeds)	1/patch
N_{germ}	Number of successfully germinated seeds	1/patch
N_{est}	Number of successfully established seedlings	1/patch
x_{ind}, y_{ind}	Random position of a mother tree on a patch	-
x_{seed}, y_{seed}	Position of a dispersed seed	-
I_{floor}	Percentage of incoming irradiance at floor	%

Table 20: *Mortality state variables.*

Symbol	Description	Unit
M_D	Mortality rate dependent on stem diameter	$1/\mathrm{yr}$
M_I	Mortality rate dependent on stem diameter increment	$1/\mathrm{yr}$
M	Mortality rate affecting individuals each time step	$1/\mathrm{yr}$
m_{frag}	Factor changing the mortality rate M due to fragmentation	-
CCA_i, $i = 1, ..., \#_{layer}$	Cumulative crown area per height layer	-
l_{min}, l_{max}	Lower and upper height layer covered by the crown of a single individual	-
R_c	Individual crowding reduction factor	-
N_C	Number of individuals dying due to crowding	$1/\mathrm{cohort}$
N_Y	Number of individuals dying due to mortality per time step	$1/\mathrm{cohort}$
N	Number of alive individuals	$1/\mathrm{cohort}$
δ_{rM}	Auxillary variable	-
N_F	Number of individuals affected by a falling tree	-

Table 21: *Light climate and growth state variables.*

Symbol	Description	Unit
$\overline{L_i}$	Individual leaf area contribution to height layer i	m^2
$\widehat{L_i}$	Patch-based leaf area index	-
I_{ind}	Incoming irradiance on top of an individual	$\mu mol_{photon}/m^2$ s
I_{leaf}	Incoming irradiance on top of the leaf surface (absorbable radiation)	$\mu mol_{photon}/m^2$ s
P_{ind}	Gross photosynthetic rate of an individual	$\mu mol_{CO_2}/$yr
P_{leaf}	Gross photosynthetic rate of a single leaf	$\mu mol_{CO_2}/m^2$ s
GPP	Gross productivity of an individual (possibly reduced)	$t_{ODM}/$yr
R_m	Maintenance respiration	$t_{ODM}/$yr
r_m	Maintenance respiration rate	1/yr

Table 22: *Water module state variables.*

Symbol	Description	Unit
Θ_{soil}	Soil water content	mm/h
IN	Interception	mm/h
RO	Run-off	mm/h
$RO_{\rightarrow}$	Surface run-off	mm/h
$RO_{\downarrow}$	Sub-surface run-off	mm/h
TR	Transpiration	mm/h
φ_W	Reduction factor of GPP due to limited soil water	-

Table 23: *Temperature state variables.*

Symbol	Description	Unit
φ_{act}	Length of vegetation period	d
φ_T	Limitation factor of GPP by temperature	-
$\varphi_{T,l}$, $\varphi_{T,h}$	Inhibition factors for low and high temperatures	-
κ_T	Factor affecting maintenance respiration rate r_M by temperature	-

Table 24: Carbon cycle state variables.

Symbol	Description	Unit
S_{dead}	Carbon stock of deadwood	$^{tC}/_{\text{patch}}$
S_{slow}	Carbon amount of slow decomposing soil stock	$^{tC}/_{\text{patch}}$
S_{fast}	Carbon amount of fast decomposing soil stock	$^{tC}/_{\text{patch}}$
S_{mort}	Carbon amount of individuals dying within the current time step	$^{tC}/_{\text{patch}}$
$t_{S_{dead}\rightarrow A}$	Transition rate of carbon from deadwood stock S_{dead} to atmosphere A	$^{tC}/_{\text{patch}}$
$t_{S_{slow}\rightarrow A}$	Transition rate of carbon from slow decomposing soil stock S_{dead} to atmosphere A	$^{tC}/_{\text{patch}}$
$t_{S_{fast}\rightarrow A}$	Transition rate of carbon from fast decomposing soil stock S_{dead} to atmosphere A	$^{tC}/_{\text{patch}}$
$t_{S_{dead}\rightarrow}$	Transition rate of carbon from deadwood stock S_{dead} to soil	$^{tC}/_{\text{patch}}$
$t_{S_{dead}\rightarrow S_{slow}}$	Transition rate of carbon from deadwood stock S_{dead} to slow decomposing soil stock S_{slow}	$^{tC}/_{\text{patch}}$
$t_{S_{dead}\rightarrow S_{fast}}$	Transition rate of carbon from deadwood stock S_{dead} to fast decomposing soil stock S_{fast}	$^{tC}/_{\text{patch}}$
NEE	Net ecosystem exchange	$^{tC}/_{\text{patch}}$
C_{GPP}	Carbon amount of gross productivity per patch	$^{tC}/_{\text{patch}}$
C_R	Carbon amount released by total respiration per patch	$^{tC}/_{\text{patch}}$

Table 25: Disturbances (fire, landslide) state variables.

Symbol	Description	Unit
N_D	Number of individuals dying due to disturbances	$^{1}/_{\text{cohort}}$
P_{F_1}, P_{F_2}, P_{F_3}, P_{F_4}	Burning probabilities for the 4 fire tolerance levels	-

G Abbreviations

**Symbol**	**Description**
ODM	Organic dry matter
CO_2	Carbon dioxide
C	Carbon
H_2O	Water
sin	Sinus function
cos	Cosinus function
$\lfloor\rfloor$	Round down
e	Exponential function
ln	Logarithm function
cf.	see
e.g.	exempli gratia (for example)
i.e.	id est (that is)
Fig.	Figure
Tab.	Table

References

Botta, A., Viovy, N., Ciais, P., Friedlingstein, P., Monfray, P., 2000. A global prognostic scheme of leaf onset using satellite data. Global Change Biology 6, 709–725.

Breuer, L., Eckhardt, K., Frede, H.G., 2003. Plant parameter values for models in temperate climates. Ecological Modelling 169, 237 – 293.

Dickinson, R.E., 1984. Modeling evapotranspiration for three-dimensional global climate models, in: Geophys. Monogr. Ser.. AGU, Washington, DC. volume 29, pp. 58–72.

Dislich, C., Günter, S.and Homeier, J., Schröder, B., Huth, A., 2009. Simulating forest dynamics of a tropical montane forest in south ecuador. Erdkunde 63, 347–364.

Fischer, R., 2010. Modellierung des Wachstums von Regenwäldern. Untersuchung der Auswirkungen von Trockenstress und Holznutzung auf den tropischen Regenwald am Beispiel des RNI Betampona (Madagaskar). Master's thesis. HTWK Leipzig.

Granier, A., BrÃ©da, N., Biron, P., Villette, S., 1999. A lumped water balance model to evaluate duration and intensity of drought constraints in forest stands. Ecological Modelling 116, 269–283.

Gutiérrez, A.G., Huth, A., 2012. Successional stages of primary temperate rainforests of chiloé island, chile. Perspectives in Plant Ecology, Evolution and Systematics 14, 243–256.

Haxeltine, A., Prentice, C.I., 1996. A general model for the light-use efficiency of primary production. functional ecology 10, 551–561.

J.Müller, M., 1996. Handbuch ausgewählter Klimastationen der Erde. Gerold Richter.

Kumagai, T., Katul, G.G., Saitoh, T.M., Sato, Y., Manfroi, O.J., Morooka, T., Ichie, T., Kuraji, K., Suzuki, M., Porporato, A., 2004. Water cycling in a bornean tropical rain forest under current and projected precipitation scenarios. Water Resour. Res. 40, W01104–.

Lambers, H., Chapin III, F., Pons, T., 2008. Plant physiological ecology. Springer.

Larcher, W., 2001. Ökophysiologie der Pflanzen. Leben, Leistung und Stressbewältigung der Pflanzen in ihrer Umwelt. UTB für Wissenschaft, Verlag Eugen Ulmer Stuttgart.

Liang, X., Lettennmaier, D., Wood, E., Burges, S.J., 1994. A simple hydrologically based model of land surface water and energy fluxes for general circulation models. Journal of Geophysical Research 99, 14415–14428.

Maidment, 1993. Handbook of hydrology.

Monsi, M., Saeki, T., 1953. Uber den lichtfaktor in den pflanzengesellschaften und seine bedeutung fur die stoffproduktion. Jpn. J. Bot 17, 22–52.

Prentice, C.I., Sykes, M.T., Cramer, W., 1993. A simulation model for the transient effects of climate change on forest landscapes. Ecological Modelling 65, 51–70.

Ryan, M.G., 1991. Effects of climate change on plant respiration. Ecological Applications 1, 157–167.

Sato, H., Itoh, A., Kohyama, T., 2007. Seib-dgvm: A new dynamic global vegetation model using a spatially explicit individual-based approach. Ecological Modelling 200, 279–307.

Schober, R., 1995. Ertragstafeln wichtiger Baumarten bei verschiedener Durchforstung. Sauerländer, Frankfurt am Main. 4 edition.

Sonntag, M., 1998. Klimaveränderung und Waldwachstum: TREEDYN3-Simulationen mit einer Analyse modellstruktureller Unsicherheiten. Ph.D. thesis. Universität Gesamthochschule Kassel.

Thornley, J.H.M., Johnson, I.R., 1990. Plant and crop modelling: a mathematical approach to plant and crop physiology. Oxford University Press.
